# The impact of a modified microbiota-accessible carbohydrate diet on gut microbiome and clinical symptoms in colorectal cancer patients following surgical resection

**DOI:** 10.3389/fmicb.2024.1282932

**Published:** 2024-02-06

**Authors:** Boyeon Kim, Jiwon Lee, Eun Sung Jung, Sunyoung Lee, Dong Ho Suh, Yu Jin Park, Jin Kim, Jung-Myun Kwak, Soohyeon Lee

**Affiliations:** ^1^Cancer Research Institute, Korea University College of Medicine, Seoul, Republic of Korea; ^2^Division of Medical Oncology and Hematology, Department of Internal Medicine, Korea University College of Medicine, Seoul, Republic of Korea; ^3^HEM Pharma Inc., Suwon, Gyeonggi, Republic of Korea; ^4^Department of Surgery, Korea University College of Medicine, Seoul, Republic of Korea

**Keywords:** modified MAC diet, colorectal cancer, microbiota, metabolites, bowel habit

## Abstract

A high-fiber diet is widely recognized for its positive effects on the gut microbiome. However, the specific impact of a high-fiber diet on the gut microbiome and bowel habits of patients with colon cancer remains poorly understood. In this study, we aimed to assess the effects of a modified microbiota-accessible carbohydrate (mMAC) diet on gut microbiota composition and clinical symptoms in colon cancer patients who underwent surgical resection. To achieve this, we enrolled 40 patients in two groups: those who received adjuvant chemotherapy and those who did not. Fecal samples were collected before and after dietary interventions for microbial and metabolite analyses. Each group was randomized in a 1: 1 ratio to follow either a 3-week conventional diet followed by a 3-week mMAC diet, or the reverse sequence. Although there were no significant differences in the microbial diversity data before and after the mMAC diet in both the non-chemotherapy and chemotherapy groups, distinct differences in gut microbial composition were revealed after the mMAC diet. Specifically, the abundance of *Prevotella*, which is associated with high-fiber diets, was further elevated with increased concentrations of acetate and propionate after the mMAC diet. Additionally, patients who experienced improved diarrhea and constipation after the mMAC diet exhibited an enrichment of beneficial bacteria and notable changes in metabolites. In conclusion, this study provides valuable insights into the potential benefits of the mMAC diet, specifically its impact on the gut microbiome and clinical symptoms in postoperative colorectal cancer (CRC) patients. These findings emphasize the potential role of a high-fiber diet in influencing the gut microbiome, and the clinical symptoms warrant further investigation.

## 1 Introduction

The human gut microbiome encompasses the genomic content of gut microbiota, consisting of a diverse array of microorganisms inhabiting the human gastrointestinal tract (GIT) (Sekirov et al., 2010; Clemente et al., 2012; Sender et al., 2016). This intricate ecosystem plays a pivotal role in various aspects of health, including digestion, immunity, and overall well-being, and even contributes to survival (Zheng et al., 2020; Wilmanski et al., 2021). These microorganisms perform distinct roles, which include the production of short-chain fatty acids (SCFAs), the metabolism of nutrients and drugs, the synthesis of vitamins, and defense against pathogens (Jandhyala et al., 2015). This complex microbial community also significantly influences the body’s immune response, potentially impacting the risk and progression of cancer (Sadrekarimi et al., 2022). Studies have revealed that alterations in the composition, diversity, and function of the gut microbiota can contribute to the development and progression of CRC (Lopez et al., 2021). Gut microbial dysbiosis, with decreasing microbial diversity, induces cancer by disrupting the balance of beneficial and harmful bacteria in the GIT. Certain bacterial species, such as *Fusobacterium nucleatum, Bacteroides fragilis, and Escherichia coli*, have been found to be more prevalent in CRC tissues than in normal tissues, suggesting that they may play a role in cancer development (Whisner and Athena Aktipis, 2019). These harmful species are known to generate carcinogenic genotoxins/metabolites, induce inflammation, and foster immunosuppressive tumor microenvironment. On the other hand, beneficial bacteria like certain strains of *Bifidobacterium* and *Lactobacillus* have shown anti-inflammatory and anti-carcinogenic effects in the colon, by reversing gut dysbiosis and activating antitumor immune system (Wong and Yu, 2023). These findings underscore the intricate connection between the gut microbiota and cancer development.

Colorectal cancer (CRC) is the third most commonly diagnosed cancer in men and the second most common cancer in women worldwide, with an estimated 1.9 million new cases and 935,000 deaths in 2020, according to the World Health Organization. Past studies have shown that colon cancer is associated with environmental factors including diet, lifestyle, smoking and gut microbiome (Song et al., 2020). Numerous studies have demonstrated a strong link between dietary patterns and the risk of colon cancer, emphasizing the significance of adopting a healthy diet to mitigate the risk of developing this condition (Davies et al., 2011; Veettil et al., 2021). Red meat, processed meat and alcohol are associated with an increased risk of CRC, particularly for left-sided CRC (Cross et al., 2010; Baena and Salinas, 2015). Higher intakes of dietary fiber, calcium, and dairy products have been associated with reduced risk of CRC incidence (Veettil et al., 2021). Although the mechanism by which diet affects cancer is not fully elucidated, the microbiome has emerged as one possible factor in this relationship. Recent research has shed light on how diet influences the gut microbiome, potentially affecting cancer development (Spencer et al., 2021).

The formation and maintenance of a healthy gut microbiome are influenced by various factors, including age, diet, lifestyle, medication use (antibiotics and non-antibiotics), and genetics (Yang et al., 2021). Among these factors, diet plays a particularly important role in shaping the composition and diversity of the gut microbes (David et al., 2014; Singh et al., 2017). A diet rich in fiber and microbiota-accessible carbohydrate (MAC), such as whole grains, fruits, and vegetables, provides essential nutrients that support the growth of beneficial bacteria in the gut. These bacteria ferment fiber and MACs to produce SCFAs, which not only provide energy for gut lining cells, but also have anti-inflammatory effects (Koh et al., 2016). For example, butyrate levels, known for their anti-inflammatory effects and role in preventing primary cancer, were increased by fiber supplements (Scharlau et al., 2009; So et al., 2018). Despite the benefits of a high-fiber diet on the gut microbiome, it may present challenges due to the rapid gas production associated with certain fiber types, leading to abdominal discomfort (El-Salhy et al., 2017). Understanding the impact and feasibility of a high-fiber diet on the gut microbiome and bowel habits is crucial for gut health comprehension and reducing the risk of diseases linked to dysbiosis, such as colon cancer.

The objective of this study was to investigate the effects of a modified microbiota-accessible carbohydrate (mMAC) diet on the gut microbiome of colon cancer patients and its potential association with clinical symptoms. Additionally, the study aimed to assess the tolerability of the mMAC diet. By examining changes in the composition and function of the gut microbiota resulting from dietary interventions, this study sought to gain insights into the potential mechanisms underlying the impact of a mMAC diet on the gut microbiome of patients with early-stage CRC.

## 2 Results

### 2.1 Patient characteristics and nutritional analysis

**Figure 1:**
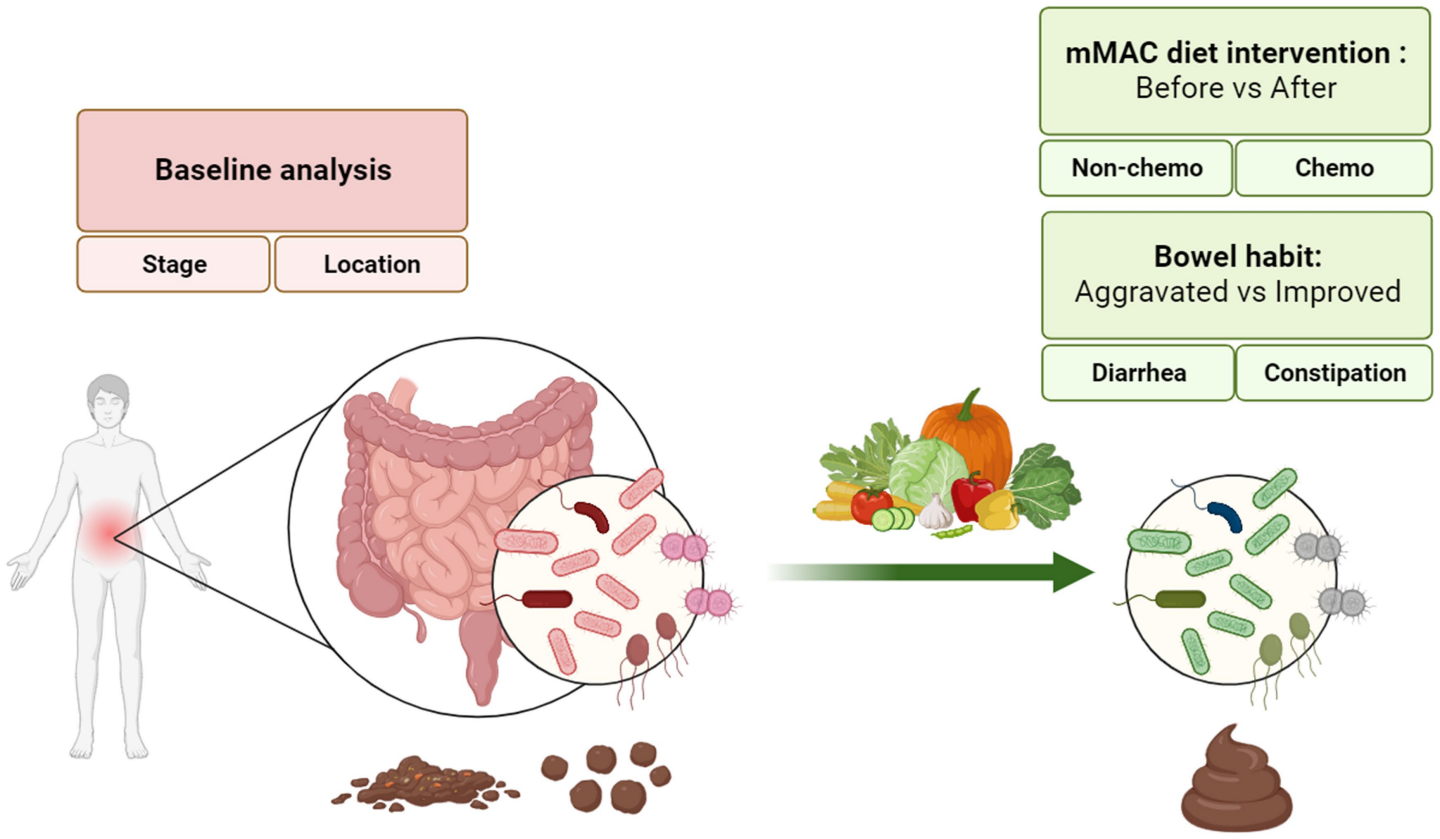
A description of the study.

Forty patients were enrolled in this study, and all participants completed the intervention. Figure 1 provides a comprehensive description of the study. Stool samples for 16S rRNA sequencing and metabolite analysis, as well as meal logs for nutritional analysis, were collected at the beginning and end of the dietary interventions (see Supplementary Figure 1A). Out of the 120 stool samples planned to be collected, 116 were successfully obtained. Among these, 113 samples met the quality control (QC) requirements for 16S rRNA sequencing, and all 116 samples underwent metabolite analysis (refer to Supplementary Figure 1B).

The baseline characteristics of the study population are summarized in Table 1, showing a median age of 61 years (range: 38–82 years). Among the patients, 29 (72.5%) had left-sided colon cancer, with 3, 21, and 16 patients having Stage I, II, and III disease, respectively. Twenty-two patients underwent chemotherapy following surgical resection, with 21 of them receiving oxaliplatin-based chemotherapy. Currently, 16 patients (40%) consumed alcohol and 5 (12.5%) were smokers.

**Table 1:** Patients’ baseline characteristics.

Baseline characteristics	Total (*N* = 40)	Chemotherapy	Non-chemotherapy	*n* (%)	22	18
Sex	Male	26 (65)	14 (60)	12 (66.7)
	Female	14 (35)	8 (40)	6 (33)
Median age (range)		61 (38 ~ 82)	62 (46 ~ 82)	58 (38 ~ 79)
Age	<60	19 (47.5)	10	9
	≧60	21 (52.5)	12	9
BMI	<25	31 (77.5)	17	14
	≧25	9 (22.5)	5	4
ECOG PS	0	10 (25)	1	9
	1	30 (75)	21	9
	2	0	0	0
Smoking	Non-smoker	19 (47.5)	11	8
	Former-smoker	16 (40)	7	9
	Current-smoker	5 (12.5)	4	1
Alcohol	Non-drinker	20 (50)	11	9
	Former-drinker	4 (10)	1	3
	Current-drinker	16 (40)	10	6
Tumor location	Right colon	11 (27.5)	7	4
	Left colon	29 (72.5)	15	14
Cancer stage	I	3 (7.5)	0	3
	II	21 (52.5)	6	15
	III	16 (40)	16	0
Chemotherapy regimen	FOLFOX	15 (37.5)	15	
	XELOX	6 (15)	6	
	Capecitabine	1 (2.5)	1	

BMI, Body Mass Index; ECOG PS, Eastern Cooperative Oncology Group Performance status.

During the analysis of food intake diaries over a 6-week study period, the overall compliance with the mMAC diet was 70.7%. Patients undergoing chemotherapy showed lower compliance (56.1%) compared to the non-chemotherapy group (86.1%). Relative to the conventional diet, there was an increase in average fiber intake during the mMAC diet period, from 22.38 g to 27.37 g (*p* < 0.0001), as well as increases in protein and fat intake, from 64.31 g to 85.97 g (*p* < 0.0001) and from 41.97 g to 50.79 g (*p* < 0.0001), respectively. Conversely, a reduction in sodium consumption was observed, from 3456.42 mg to 3245.94 mg (*p* = 0.0296) (see Supplementary Figure 2). The percentage of diet composition consumed relative to the recommended daily intake is detailed in Supplementary Table 1.

### 2.2 Analysis of gut microbiota diversity and composition according to the baseline characteristics

Initially, we analyzed the microbial diversity and composition in relation to cancer stage and location. The cancer stage analysis involved classifying patients into either stage I and II or stage III. While little difference in alpha diversity was noted between these two groups, a significant difference in beta diversity was identified by weighted Unifrac distance (*p* = 0.046) (refer to Supplementary Figures 3A,B). The LEfSe analysis, conducted to identify distinct microorganisms for each group, revealed that *Rothia*, *Coprobacillus*, *Solobacterium*, [*Eubacterium*]_*eligens*_*group*, *Veillonella*, and *Lactobacillus* were significantly more abundant in stage III patients (see Supplementary Figures 3C–E).

Regarding the analysis based on location, no significant differences were found in alpha diversity between left and right-sided colon cancer (Supplementary Figure 4A). However, principal coordinate analysis (PCoA) using weighted Unifrac distance indicated a significant difference in beta diversity (*p* = 0.023) (Supplementary Figure 4B). In patients who underwent surgery for left-sided colon cancer, *Sellimonas*, [*Eubacterium*]_*eligens*_*group*, and *Anaerofustis* were significantly enriched. Conversely, in patients with right-sided cancer who underwent surgery, there was a significant increase in *Peptoniphilus*, *Lachnospiraceae*_*UCG*-*010*, [*Ruminococcus*]_*torques*_*group*, *Dorea*, *Blautia*, and *Holdemanella* (Supplementary Figures 4C–E).

### 2.3 The effect of mMAC diet on non-chemotherapy and chemotherapy groups

**Figure 2:**
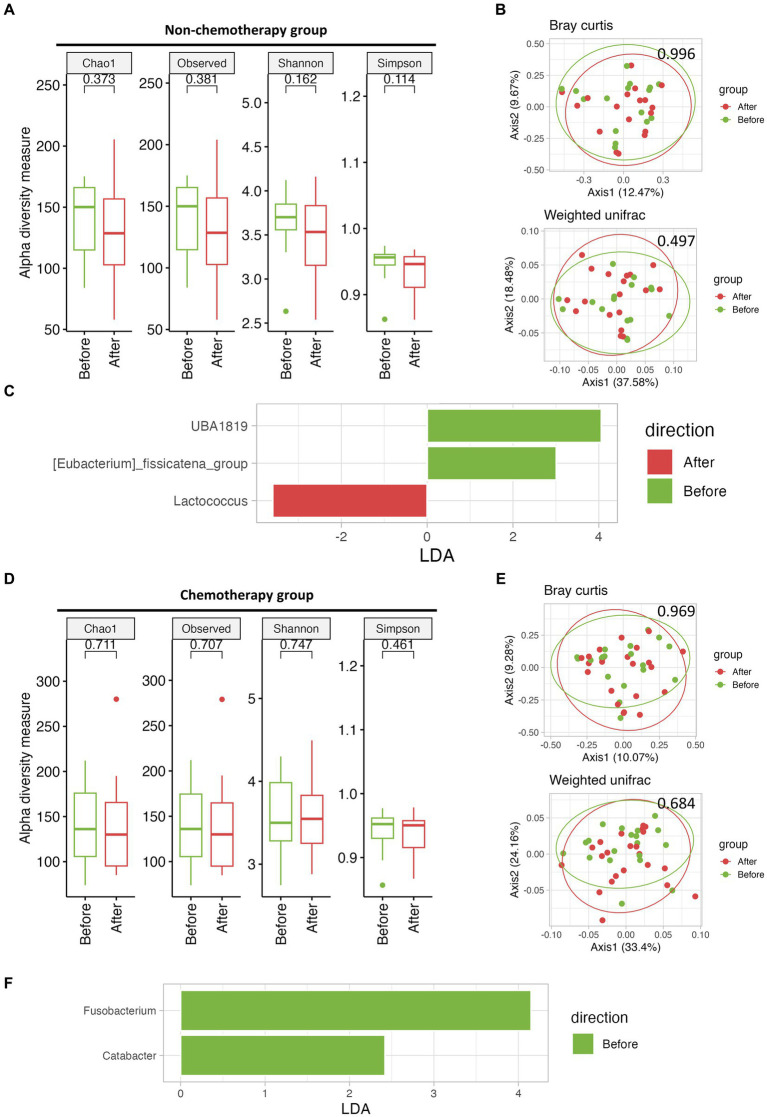
The effect of mMAC diet on microbial diversity and composition in non-chemotherapy and chemotherapy groups. **(A)** Alpha-diversity, as measured by the Chao1, Observed ASVs, Shannon, and Simpson indices, is depicted for the non-chemotherapy group both Before (green) and After (red) the mMAC diet. **(B)** PCoA plots represent beta-diversity, specifically Bray Curtis (top) and weighted Unifrac (bottom). In these plots, each point symbolizes a single sample, color-coded as Before (green) and After (red) the mMAC diet in the non-chemotherapy group. **(C)** Genus-level LEfSe analysis identified taxa with differential abundance Before (green) and After (red) the mMAC diet in the non-chemotherapy group. **(D)** Alpha-diversity metrics are plotted for the chemotherapy group Before (green) and After (red) the mMAC diet. **(E)** PCoA plots for this group also represent Bray Curtis (top) and weighted Unifrac (bottom) beta-diversity. **(F)** The genus-level LEfSe analysis in the chemotherapy group revealed significantly abundant taxa Before (green) the mMAC diet.

To evaluate the effects of a high-fiber diet on microbiome differences, we compared the diversity and microbial composition before and after the mMAC diet. Due to the compliance gap between the non-chemotherapy and chemotherapy groups, each group was analyzed separately. No significant differences were observed in alpha and beta diversity Before and After the mMAC diet in either group (Figures 2A,B,D,E). However, LEfSe analysis revealed distinct microbial differences between the Before and After mMAC diet groups. In the non-chemotherapy group, post-diet samples showed significant enrichment for *Lactococcus* while *UBA1819* and [*Eubacterium*]_*fissicatena*_*group* were decreased (Figure 2C and Supplementary Figures 5A,B). In the chemotherapy group, *Fusobacterium* and *Catabacter* were decreased in the After group (Figure 2F and Supplementary Figure 5C).

Precise LEfSe analysis up to the species level confirmed that the post-mMAC diet samples had an increased presence of several species belonging to the genera *Lactococcus*, *Lactobacillus*, and *Bifidobacteria* (Supplementary Figures 5D–I).

### 2.4 Short chain fatty acid analysis according to three dominant bacteria for enterotypes: *Prevotella*, *Bacteroides* and *Ruminococcus*

The human gut microbiome can be classified into one of three traditionally reported enterotypes of bacterial patterns: *Bacteroides* (enterotype 1), *Prevotella* (enterotype 2), and *Ruminococcus* (enterotype 3) (Arumugam et al., 2011; Gorvitovskaia et al., 2016).

**Figure 3:**
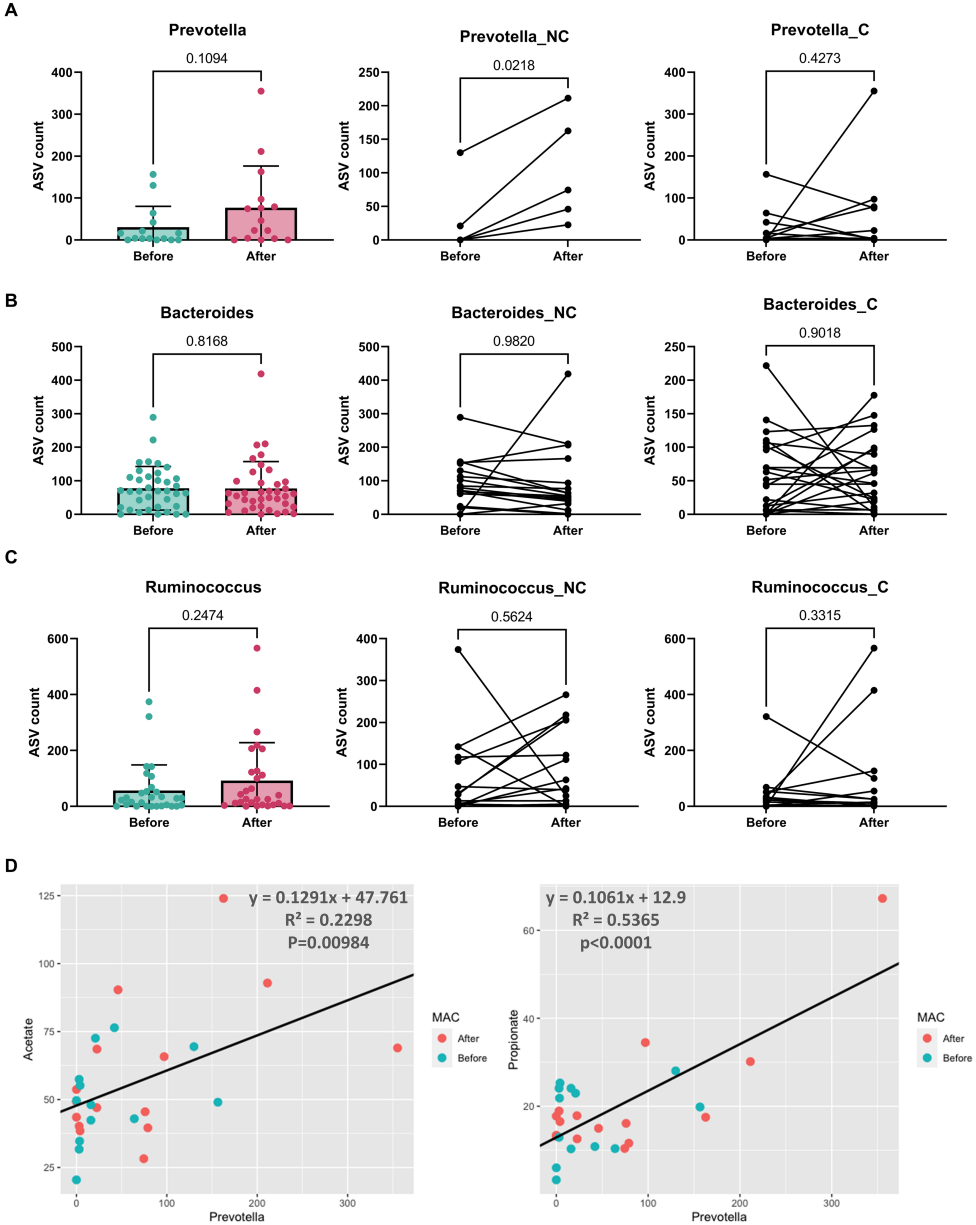
The abundance and correlation analysis of three enterotype dominant taxa and metabolites. **(A–C)** The abundance of *Prevotella* **(A)**, *Bacteroides* **(B)**, and *Ruminococcus* **(C)** in patients Before (green) and After (red) the mMAC diet. The comparison includes the entire patient cohort (left), the non-chemotherapy group (middle), and the chemotherapy group (right). **(D)** A correlation analysis was conducted between *Prevotella* and SCFAs (acetate and propionate). The analysis utilized *Prevotella*’s amplicon sequence variant (ASV) count and the concentration (μmol/g) of SCFAs. A black line on each graph represents the trend line for all samples, with the corresponding equation, R-squared (*R*^2^) values, and *p*-values indicated.

We thus examined the abundance of *Bacteroides*, *Prevotella*, and *Ruminococcus* in individuals before and after adopting the mMAC diet. Notably, the abundance of *Prevotella* increased in the after group, particularly among those not undergoing chemotherapy (*p* = 0.0218). In contrast, the abundances of *Bacteroides* and *Ruminococcus* showed no significant differences (Figures 3A–C).

Furthermore, a positive correlation was observed between the abundance of *Prevotella* and two SCFAs, acetate and propionate, with *R*^2^ values of 0.2298 and 0.5365, respectively (*p* = 0.00984 and *p* < 0.0001) (Figure 3D). Butyrate showed a weak positive correlation (*R*^2^ = 0.021, *p* = 0.4615) (Supplementary Figure 6A). The correlations between *Bacteroides* or *Ruminococcus* and SCFAs were generally negative but weak, with only the correlation between *Bacteroides* and valerate reaching statistical significance (*R*^2^ = 0.0995, *p* = 0.007812) (Supplementary Figures 6B,C).

### 2.5 The impact of mMAC diet on bowel habits and its association with the gut microbiome

**Figure 4:**
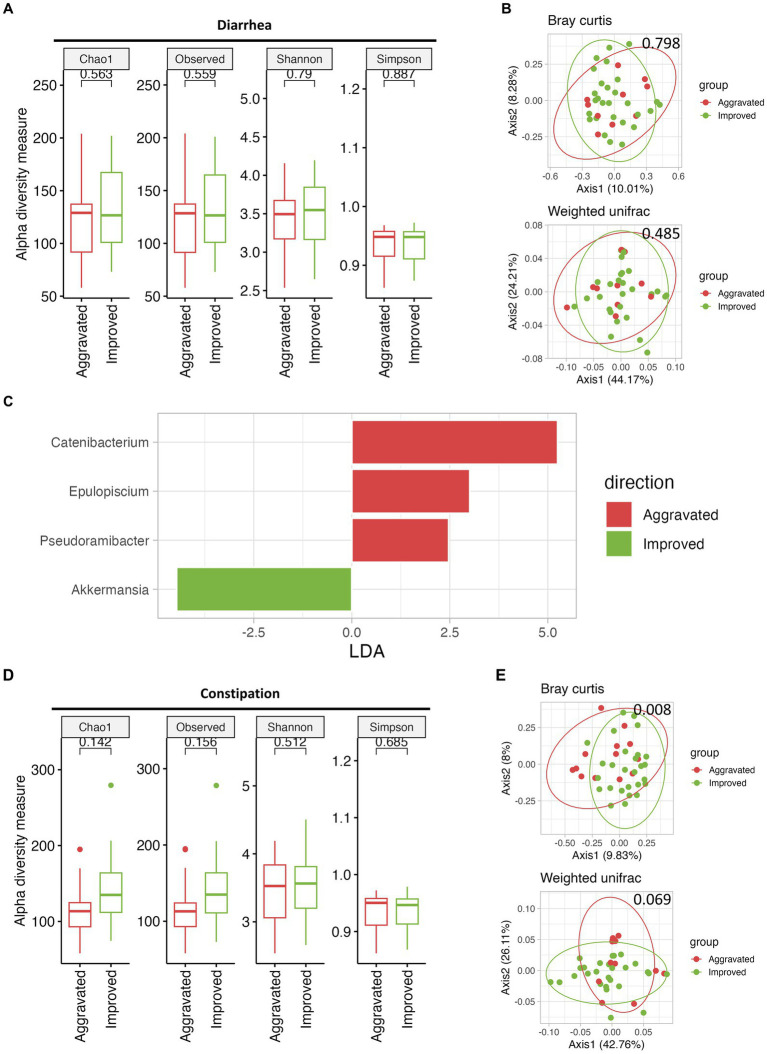

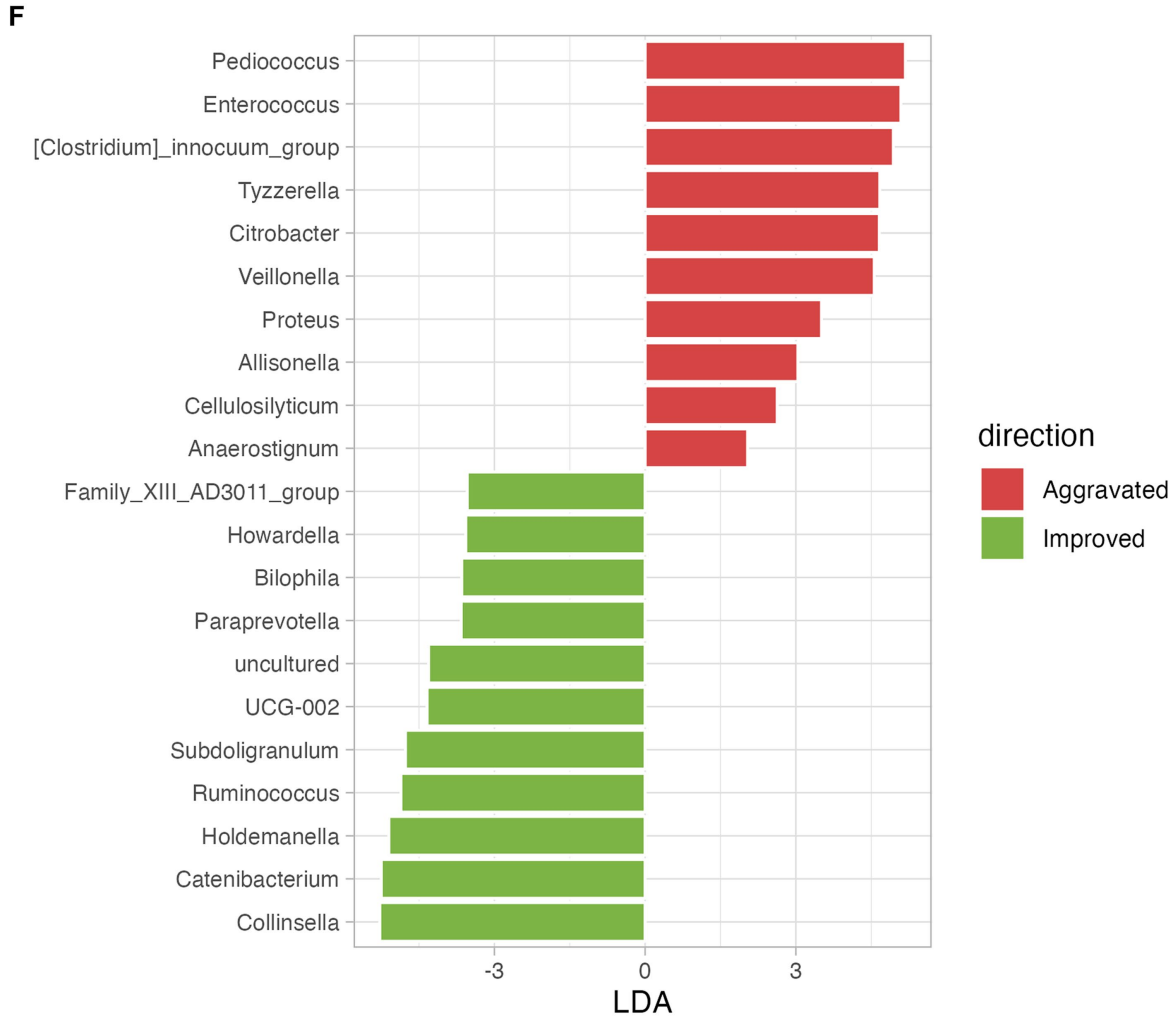
The effect of mMAC diet on microbial diversity and composition according to gastrointestinal (GI) symptom. **(A)** Alpha-diversity measurements are depicted for the Aggravated (red) and Improved (green) diarrhea groups following the mMAC diet. **(B)** PCoA plots illustrate beta-diversity using Bray Curtis (top) and weighted Unifrac (bottom) methods. **(C)** Genus-level LEfSe analysis identified significantly abundant taxa in both the Aggravated (red) and Improved (green) diarrhea groups. **(D)** Alpha-diversity plots are shown for the Aggravated (red) and Improved (green) constipation groups post-mMAC diet. **(E)** PCoA plots again represent Bray Curtis (top) and weighted Unifrac (bottom) beta-diversity for these groups. **(F)** The genus-level LEfSe analysis reveals significantly abundant taxa in the Aggravated (red) and Improved (green) constipation groups.

Following the mMAC diet, 28 and 27 patients reported improvement in diarrhea and constipation, respectively, while 10 and 13 patients experienced worsening symptoms. To assess the relationship between the gut microbiome and symptom relief, we compared the microbial diversity and SCFAs in patients with either aggravated or improved diarrhea and constipation post-mMAC diet consumption. Notably, there were no significant differences in alpha and beta diversity between the Improved and Aggravated diarrhea groups (Figures 4A,B). However, the Chao1 and Observed ASV indices in the Improved constipation group tended to be higher compared to the Aggravated group (Figure 4D). Additionally, Bray Curtis and weighted Unifrac beta diversity indices showed significant differences between these two groups (*p* = 0.008 and *p* = 0.069) (Figure 4E). LEfSE analysis revealed an association between *Akkermansia* and the Improved diarrhea group (Figure 4C and Supplementary Figures 7A,B). Furthermore, the genera *Family*_*XIII*_*AD3011*_*group*, *Howardella*, *Bilophila*, *Paraprevotella*, *UCG*-*002*, *Subdoligranulum*, *Ruminococcus*, *Holdemanella*, *Catenibacterium*, and *Collinsella* were associated with Improved constipation (Figure 4F and Supplementary Figures 7D,E). In the metabolite analysis, levels of acetate and propionate were slightly increased in the Improved diarrhea group (*p* = 0.0945 for both) and valerate was significantly increased in the Improved constipation group (*p* = 0.0118) (Supplementary Figures 7C,F).

## 3 Discussion

This study examined the impact of the mMAC diet on the gut microbial composition of patients with early-stage CRC. Our primary objective was to ascertain any differences in the microbiome before and after the mMAC diet, considering factors such as chemotherapy and bowel habits. The findings revealed that a three-week mMAC diet regimen did not significantly alter the alpha and beta diversities of the gut microbiome in both the non-chemotherapy and chemotherapy groups. However, notable microbial differences were observed in both groups post-diet. Importantly, beneficial intestinal bacteria, including *Lactobacillus* and *Bifidobacterium*, were found to be enriched following the mMAC diet. Additionally, a significant increase in the abundance of *Prevotella* was observed in the non-chemotherapy group after following the mMAC diet. This increase in *Prevotella* was positively correlated with higher levels of SCFAs such as acetate and propionate.

### 3.1 Gut microbiome and tumor stages and locations

Previous studies have demonstrated variations in bacterial populations across different sites and stages of CRC (Nakatsu et al., 2015; Mima et al., 2016a). Distinct microbiome profiles have been observed between left-sided and right-sided CRC (Zhong et al., 2020; Miyake et al., 2021). However, the results obtained among various studies did not align or prove to be reproducible. It is likely that various factors influencing the microbiome played a role, as evidenced by a study that observed differences in microbiomes among different geographical regions in patients with CRC (Zhong et al., 2020). Among harmful microorganisms, *Fusobacterium nucleatum*, in particular, has been implicated in the carcinogenesis of right-sided CRC (Sheng et al., 2019; Yachida et al., 2019). Additionally, the levels of *Fusobacterium nucleatum* have been positively associated with advanced stages of CRC (Mima et al., 2016b). In our study, however, we observed no significant difference in *Fusobacterium nucleatum* levels across different stages and locations of CRC (data not shown). Our analysis was conducted using fecal samples, and it’s possible, as noted in prior research, that the quantity of *Fusobacterium nucleatum* in fecal samples was insufficient compared to that in tumor tissues (Sheng et al., 2019).

In contrast to the majority of previous studies that analyzed the microbiome before surgical resection, we compared the microbiome of patients who underwent resection for CRC. We found that beta-diversity was significantly different, with distinct microbial profiles emerging after surgical resection of right-sided CRC compared to left-sided CRC. A previous study reported lower levels of *Gemellaceae* in patients who underwent right CRC resection, while the *Firmicutes* phylum was found to be abundant in those who underwent sigmoid colon cancer resection (Suga et al., 2022). Taken together, our results underscore the difference in microbiome composition based on the location of resection.

We also found distinct microbial profiles between Stages. Increased levels of *Rothia*, *Coprobacillus*, *Solobacterium*, [*Eubacterium*]_*eligens*_*group*, *Veillonella*, and *Lactobacillus* were found in patients with stage III CRC. *Rothia dentocariosa* and *Veillonella*, members of the oral microbiota, have been suggested to contribute to the pathogenesis of inflammatory bowel disease (IBD) and CRC (Koliarakis et al., 2019). Conversely, *Lactobacillus brevis* has been associated with suppressing the progression of CRC (Sakatani et al., 2016). Further research is necessary to explore the interactions between these beneficial and harmful bacteria and to understand how the overall microbial ecosystem influences the development and recurrence of CRC.

### 3.2 The impact of mMAC diet on gut microbiome and SCFA

MACs are carbohydrates that can be fermented by gut microbes, serving as the main source of energy. They are used in the production of various metabolites, including SCFAs, which play crucial roles in maintaining gut health and influencing various physiological processes MAC as novel gut microbiome modulators in noncommunicable diseases. In previous studies, reduced MAC intake was associated with decreased bacterial diversity leading to dysbiosis (Sonnenburg et al., 2016; Fehlbaum et al., 2018). This, in turn, suggests that a MAC-rich diet may increase bacterial diversity. But, clinical trials showed contradictory results and some have even demonstrated decreased alpha diversity after the use of MAC (Cantu-Jungles and Hamaker, 2023). This could be attributed to various factors that have been suggested to affect diversity, such as the number of microbes sharing the dietary fiber substrate, the initial abundance of bacterial taxa, and the composition of MACs. Despite the high fermentable fiber content of the mMAC diet used in this study, no significant changes in microbial diversity were observed before and after the MAC diet. It is possible that the three-week duration of the dietary intervention was not long enough to affect a notable increase in microbial diversity.

In line with prior research (Wu and Kasper, 2020), we noted an increase in the abundance of *Prevotella* following the mMAC diet, especially in the non-chemotherapy group. This increase was positively correlated with two SCFAs, acetate and propionate. Up to now, the association of dietary fibers with SCFAs has not been fully elucidated (Vinelli et al., 2022). Further investigation is essential to elucidate the interplay among dietary fiber, SCFAs, and their effects on patients with CRC.

### 3.3 Feasibility and changes in bowel habits with the mMAC diet

In this study, we discovered that postoperative CRC patients are able to tolerate a high-fiber diet, specifically the mMAC diet, which includes a daily intake of 30 grams of fiber. Contrary to the initial concerns about potential discomfort, the compliance rate for the mMAC diet was notably high at 70% (Ioniță-Mîndrican et al., 2022). 28 patients (73.7%) experienced an improvement in diarrhea, while 27 patients (67.5%) showed improvement in constipation after mMAC diet. Previous studies have demonstrated that the gut microbiota, especially dysbiosis, significantly influences gastrointestinal symptoms such as diarrhea and constipation (Ohkusa et al., 2019; Li et al., 2021). Modulating the gut microbiota through interventions such as diet control, probiotics, prebiotics, and fecal transplantation has been attempted to control GI symptoms. Our study found distinct microbial profiles in improved groups compared to aggravated groups. Patients who experienced improvements in constipation showed a significant trend toward an increase in bacteria, such as *Paraprevotella*, Subdoligranulum and *Holdemanella*. Patients who experienced improved diarrhea showed higher *Akkermansia.* While the role of *Akkermansia muciniphila* in IBD is still a subject of debate, some studies have suggested its potential in regulating immune cells (Zhang et al., 2021). Given that our study included patients undergoing adjuvant chemotherapy, caution is needed when interpreting the results of the microbiome, as they may not solely be attributed to the effects of the mMAC diet. In subsequent studies, it is necessary to elucidate the respective impacts of cancer treatment and the mMAC diet on the gut microbiome in CRC patients.

### 3.4 Limitations of this study

Despite these intriguing findings, our study has several limitations. First, we did not assess the impact of the mMAC diet on critical clinical outcomes, such as cancer recurrence or survival, focusing solely on patients with early stage CRC within a limited timeframe. Secondly, the crossover of the dietary intervention may have influenced outcomes, as the former diet might have a carry-over effect. Thirdly, the small sample size limited the comprehensive assessment of changes in gut microbiome diversity, rendering the observed results exploratory in nature.

### 3.5 Conclusion

In conclusion, this study provides valuable insights into the potential benefits of the mMAC diet, specifically its impact on the gut microbiome and clinical symptoms in postoperative CRC patients. Although the mMAC diet did not lead to significant diversity, notable trends in microbial composition were observed in patients with improved diarrhea symptoms. These findings emphasize the potential role of a high-fiber diet in influencing the microbiome, and the clinical symptoms warrant further investigation.

## 4 Patients and methods

We conducted a prospective, randomized, crossover trial with dietary intervention in patients with localized CRC patients who underwent surgery. Written informed consent was obtained from all patients. This study was approved by our Institutional Review Board (2021AN0489) and was registered at ClinicalTrials.gov (NCT05039060). The trial procedures were performed in accordance with the Declaration of Helsinki and Guidelines for Good Clinical Practice.

### 4.1 Patient selection

The patients were enrolled in the group receiving adjuvant chemotherapy for half of the patients (chemotherapy group) and the other half for not receiving adjuvant chemotherapy (non-chemotherapy group) according to the postoperative stage from Korea University Anam Hospital. Each group was randomized separately in a 1: 1 ratio to start with one of two diet sequences: either a conventional diet followed by an mMAC diet or an mMAC diet followed by a conventional diet, with each diet lasting 3 weeks (see Supplementary Figure 1A). The inclusion criteria were Stage I to III CRC patients who underwent complete curative surgical resection, did not have an ileostomy, were appropriate for a solid diet, were above 19 years of age, and had Eastern Cooperative Oncology Group (ECOG) scores of 0 to 2. The exclusion criteria were Stage IV CRC, a history of antibiotics within 1 month, a history of food allergy, uncontrolled diabetes mellitus (HbA1c > 8.0 g/dL), and other medical conditions that impair the ability to adhere to diet intervention.

### 4.2 Subject characterization

Demographic and laboratory information were obtained for all subjects; including Sex, Age, BMI, ECOG performance status, body mass index, Tumor location, Cancer stage, adjuvant chemotherapy regimen. The clinical symptoms, including diarrhea and constipation, were assessed using The European Organization for Research and Treatment of Cancer (EORTC) Quality of Life Questionnaire, QLQ-C30 (version 3.0).

### 4.3 Dietary intervention

In each treatment group, the participants were further divided into two groups through random assignment and assigned to a 3-week mMAC diet group or a conventional diet group, followed by a diet switch for the next 3 weeks. During the 6-week study period, the patients kept records of their diets and defecation in their diaries. At the end of the study, diaries were collected to evaluate the participants’ food intake and defecation status at the end of the study (Supplementary Figure 1).

The mMAC diet was designed to adapt to a high-fiber diet including 30 g of dietary fiber daily and delivered to the patient’s home for 3 weeks as a meal kit supported by Dr. Kitchen Corp (Supplementary Table 2).

### 4.4 Stool sample collection and DNA extraction

All stool samples were kept in a participant’s home freezer (−20°C) wrapped in ice packs until they were transferred on ice to the research laboratory and stored in a deep freezer (−80°C). After thawing, each sample was manually homogenized using a sterile tip and small aliquots of the sample were collected in a 2.0 mL microtube (0.2 g) for microbiome and metabolome analysis. Fecal bacterial genomic DNA extraction was performed using the Mag-Bind^®^ Universal Pathogen Kit (Omega Bio-tek, Norcross, GA, US). Detailed stool DNA extraction and next generation sequencing methods are described in the Supplementary Methods section.

### 4.5 Next generation sequencing and metabolite analysis

Next generation sequencing (NGS) was performed to analyze the composition and diversity of the gut microbiota. Headspace sampler-gas chromatography-flame ionization detector (HSS-GC-FID) analysis was performed for microbial metabolite analysis. Detailed methods are described in the Supplementary Methods section.

### 4.6 Microbial diversity, taxonomic profiling and statistical analysis

Alpha diversity was quantified as the number of observed ASVs, Chao1, Shannon, and Simpson indices. Wilcoxon signed-rank tests were used to evaluate the differences in diversity among samples. Bray Curtis and weighted Unifrac distance matrices for beta diversity were obtained and *q*-values were calculated using QIIME2. Subsequently, these matrices were imported into R to obtain principal coordinate analysis (PCoA) plots. The Linear Discriminant Analysis (LDA) Effect Size (LEfSe) algorithm was applied to identify taxonomic biomarkers in genera and species level. Default parameters were used for significance (*p* < 0.05) and the linear discriminant analysis threshold (LDA score > 2.0). The Mann–Whitney test was applied to compare individual microbiota and SCFAs. SCFAs and microbial correlation was confirmed by linear regression with *R*^2^ and value of *p*.

All analyses were performed using R packages (Qiime2R, Microbial, Microbiomeutilities) and GraphPad Prism (version 9.0).

## Data availability statement

The datasets presented in this study can be found in online repositories. The names of the repository/repositories and accession number(s) can be found at: https://www.ncbi.nlm.nih.gov/, PRJNA1008784.

## Ethics statement

The studies involving humans were approved by the Korea University Institutional Review Board. The studies were conducted in accordance with the local legislation and institutional requirements. The participants provided their written informed consent to participate in this study.

## Author contributions

BK: Formal analysis, Investigation, Methodology, Visualization, Writing – original draft. JL: Data curation, Resources, Writing – original draft, Project administration. ESJ: Methodology, Writing – review & editing. SuL: Formal analysis, Writing – review & editing. DHS: Methodology, Writing – review & editing. YJP: Formal analysis, Writing – review & editing. JK: Resources, Writing – review & editing. J-MK: Resources, Writing – review & editing. SoL: Conceptualization, Project administration, Supervision, Writing – review & editing.
