# Supplementary material for: The impact of a modified microbiota-accessible carbohydrate diet on gut microbiome and clinical symptoms in colorectal cancer patients following surgical resection
Source: Front Microbiol. 2024 Feb 6;15:1282932. doi: 10.3389/fmicb.2024.1282932 (PMC10877053; doi:10.3389/fmicb.2024.1282932)
Supplement: Supplementary file 1 [file Table_1.DOCX]

Supplementary Material

# Supplementary Methods

## DNA extraction and next generation sequencing (NGS) analysis

The fecal sample was suspended in 275 μL of SLX-Mlus Buffer, followed by bead beating in a mixermill MM400 (Retsch, Haan, Nordrhein-Westfalen, Germany) with further isolating, cleaning, and eluting procedures followed the manufacturer’s protocols. Ribosomal RNA gene amplicons for the Illumina Miseq System (Illumina, San Diego, California, US) describe a method for preparing samples for sequencing the variable V3-V4 regions of the 16S rRNA gene. The extracted fecal microbial DNA was amplified with 16S Amplicon PCR Forward Primer (5’-TCGTCGGCAGCGTCAGATGTGTATAAGAGACAGCCTACGGGNGGCWGCAG-3’) and 16S Amplicon PCR Reverse Primer (5’-GTCTCGTGGGCTCGGAGATGTGTATAAGAGACAGGACTACHVGGGTATCTAATCC-3’). These amplicon primers, 2× KAPA HiFi HotStart ReadyMix (Roche, Basel, Basel-Stadt, Switzerland) and DNA were generated by PCR under conditions of 3 min at 95 °C, followed by 25 cycles at 95 °C for 30 s, annealing at 55 °C for 30 s, extension at 72 °C for 30 s and a final extension at 72 °C for 5 min. Subsequently sample DNAs were cleaned with HiAccuBead (AccuGene, Incheon, South Korea) and a magnetic stand. The Index PCR was performed by a using the IDT indexing primer (Integrated DNA technologies, Coralville, Iowa, US) for Illumina Miseq System, 2× KAPA HiFi HotStart ReadyMix, and PCR grade water. PCR was carried out in a 95 °C for 3 min. 8 cycles of 95 °C for 30 s, 55 °C for 30 s, 72 °C for 30 s, then 72 °C for 5 min and held at 4 °C for PCR reaction. After clean-up step, the concentration of libraries was verified using the Qubit 4.0 (ThermoFisher Scientific, Waltham, Massachusetts, US) with 1× dsDNA HS assay solution (ThermoFisher Scientific, Waltham, Massachusetts, US) and sequenced using Illumina Miseq system. Reads were sorted using the unique barcodes for each PCR product. The barcode, linker and primer sequences were then removed from the original sequencing reads. The sequencing results was analyzed using Qiime2 bioinformatics pipeline and taxonomic assignment was performed with Silva reference database at a sampling depth of 13000.

## Headspace sampler-gas chromatography-flame ionization detector (HSS-GC-FID) analysis

All short-chain fatty acids (SCFAs) were extracted with 0.2 g of fecal sample in 1 mL of dH2O. After vortexing, all samples were centrifuged at 13,000 rpm for 3 min at room temperature. The supernatant of the centrifuged samples (150 μL) was transferred to 10 mL of screw cap vial with 150 μL of GC buffer solution, which contains (NH4)2SO4, NaH2PO4, and 2-ethylbutric acid were added as internal standard (Zhang et al., 2018). HSS-GC-FID analysis was performed by Agilent 7890B GC system equipped with a 7697A headspace sampler and FID (Agilent Technologies, USA). An HP-innowax capillary column (30 m x 0.32 mm i.d. x 0.50 μL film thickness; Agilent) was used with constant flow of nitrogen as the carrier gas. The operating conditions were as follows; oven temperature, 85 °C; loop temperature 90 °C; transfer lines, 100 °C; FID temperature 250 °C; column temperature was initially at 60 °C, raised to 140 °C at 30 °C/min, then raised to 170 °C at 30 °C, and finally to 180 °C at 40 °C and held for 0.75 min. Data acquisition and operation processing were conducted using ChemStation software (Agilent Technologies). SCFAs were identified and quantified using standard compounds.

# Supplementary Figures

## Supplementary Figure 1


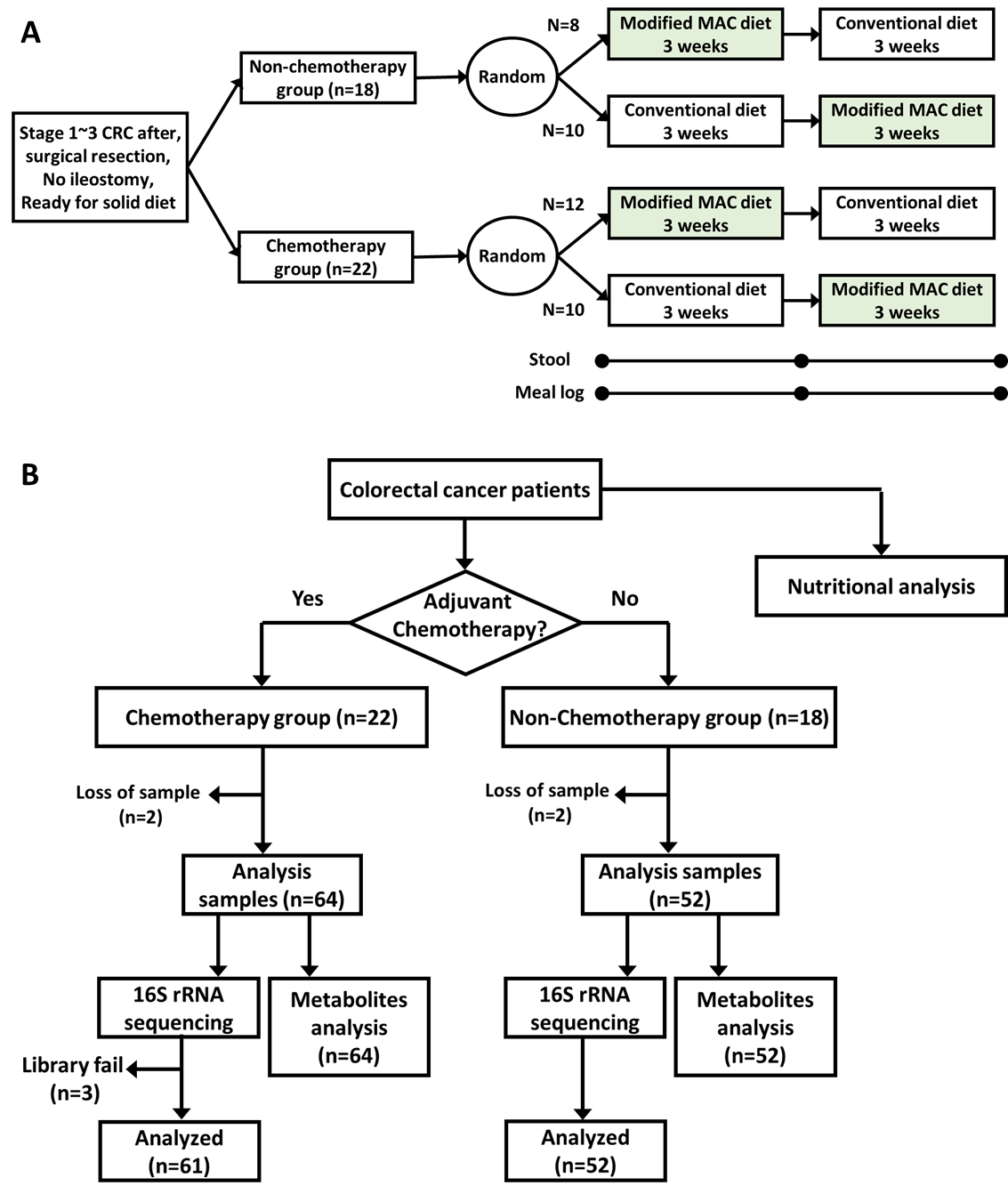


**Figure 1. A schematic diagram of the study.** (A) Schematic representation of a prospective, randomized, crossover trial with diet intervention on localized colorectal cancer patients who underwent surgery. (B) Overview of the 16S rRNA sequencing and metabolites analysis workflow.

## Supplementary Figure 2


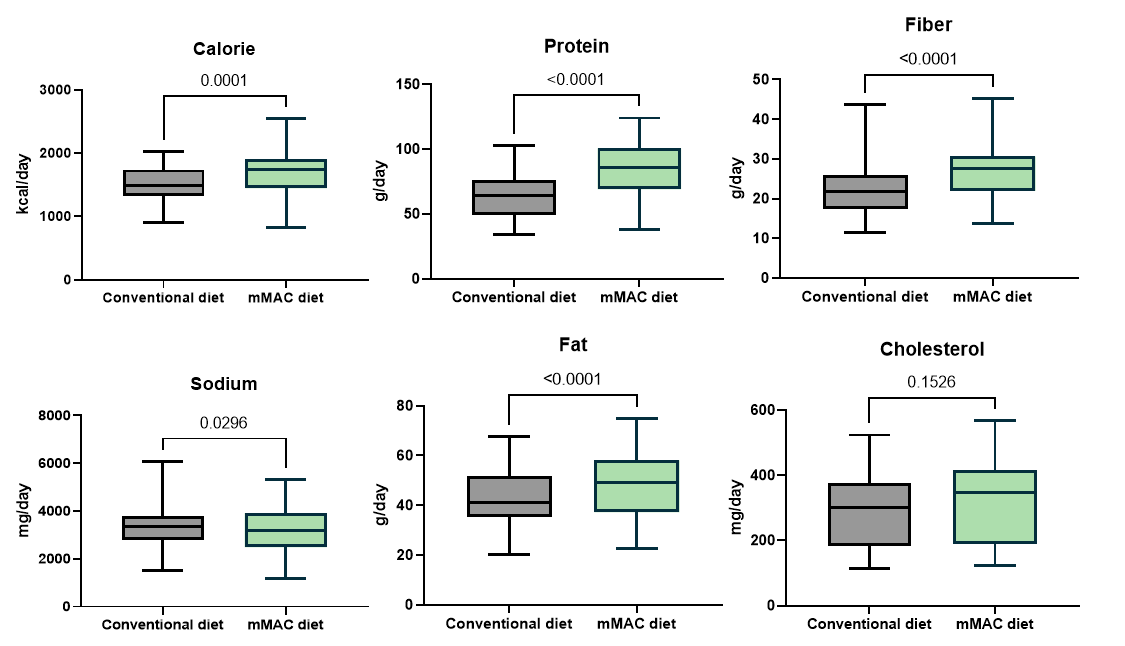


**Supplementary figure 1.** Nutritional analysis (A) Box and whisker plots represent for nutrients ingested during diet interventions.

## Supplementary Figure 3


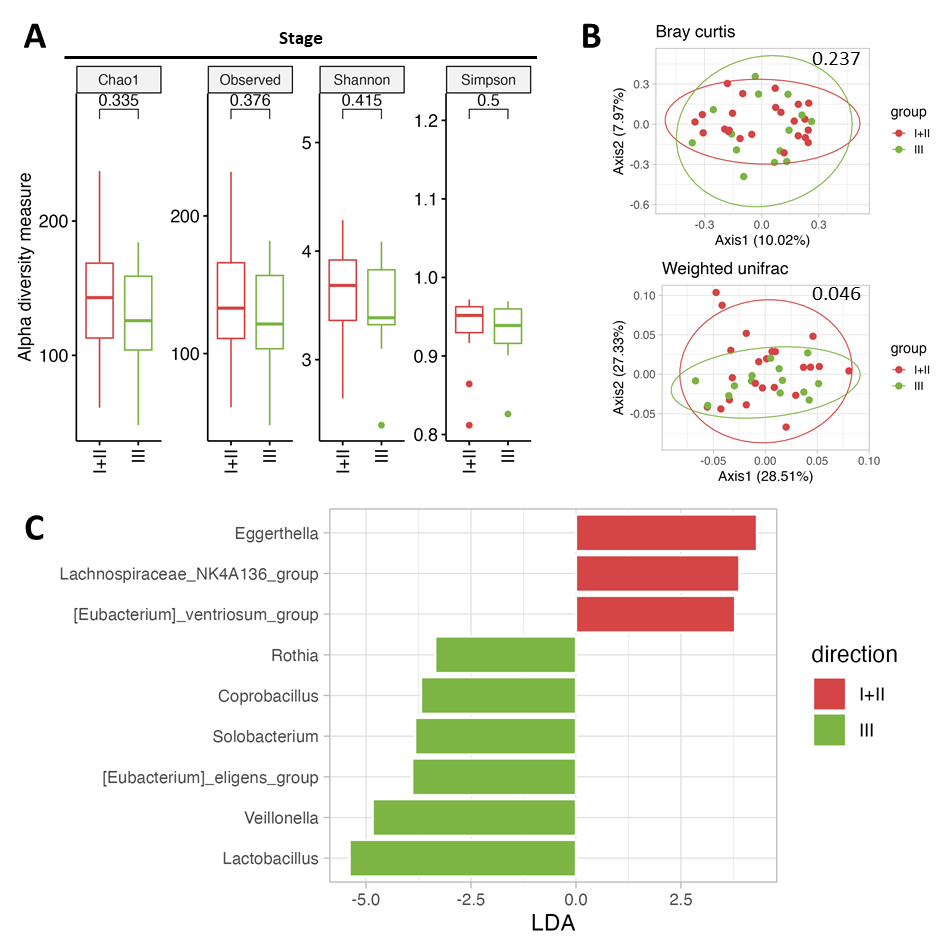


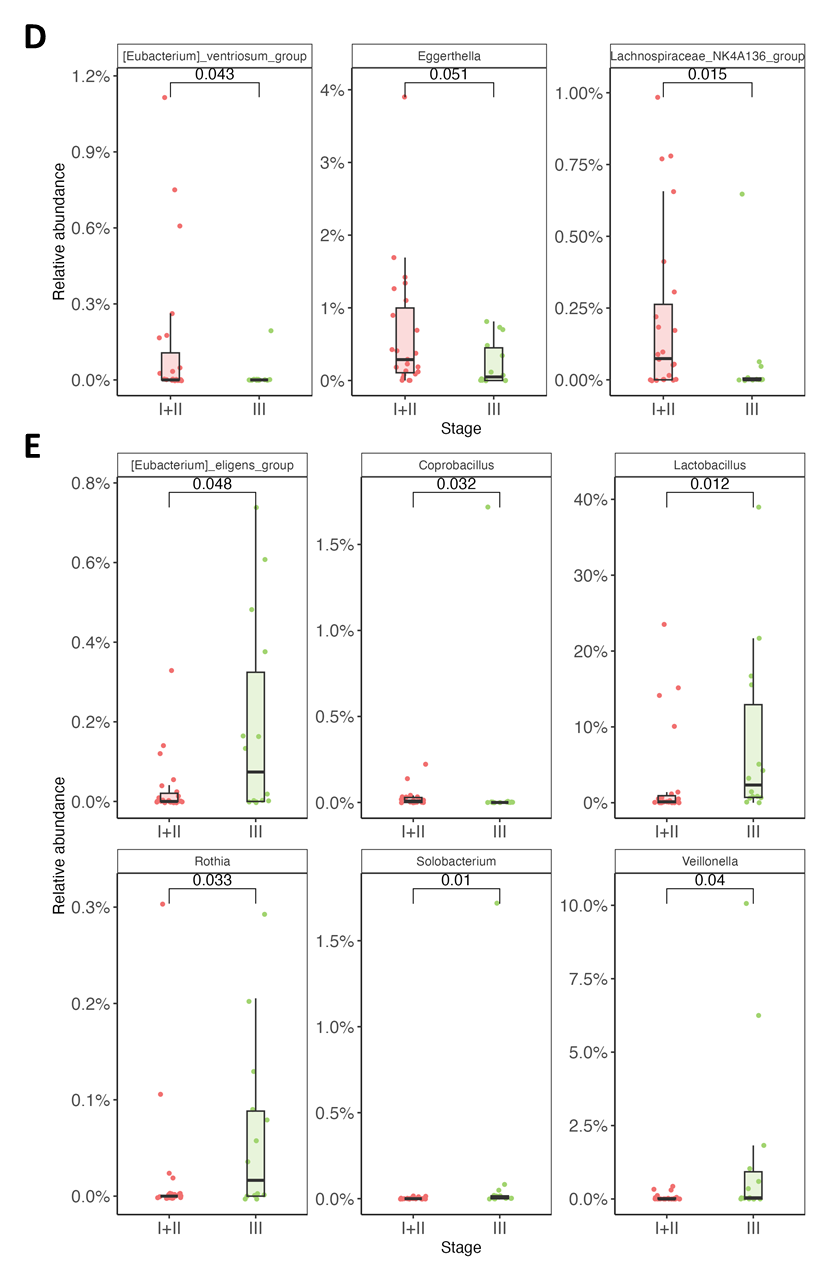


**Supplementary figure 3.** The effect of tumor stage on microbial diversity and composition. (A) Alpha-diversity, as measured by Chao1, Observed ASVs, Shannon, and Simpson indices, is illustrated for patients in stage I and II (red) compared to those in stage III (green). (B) PCoA plots are shown for Bray Curtis (top) and weighted Unifrac (bottom), representing beta-diversity. Each point in these plots corresponds to a single sample, color-coded for stage I and II (red) and stage III (green) colon cancer groups. (C) LEfSe analysis identified significantly abundant genus-level taxa between group I and II (red) and group III (green). (D-E) Individual box plots for each sample are provided, with dots signifying differences in microorganisms as identified through the LEfSe analysis.

## Supplementary Figure 4


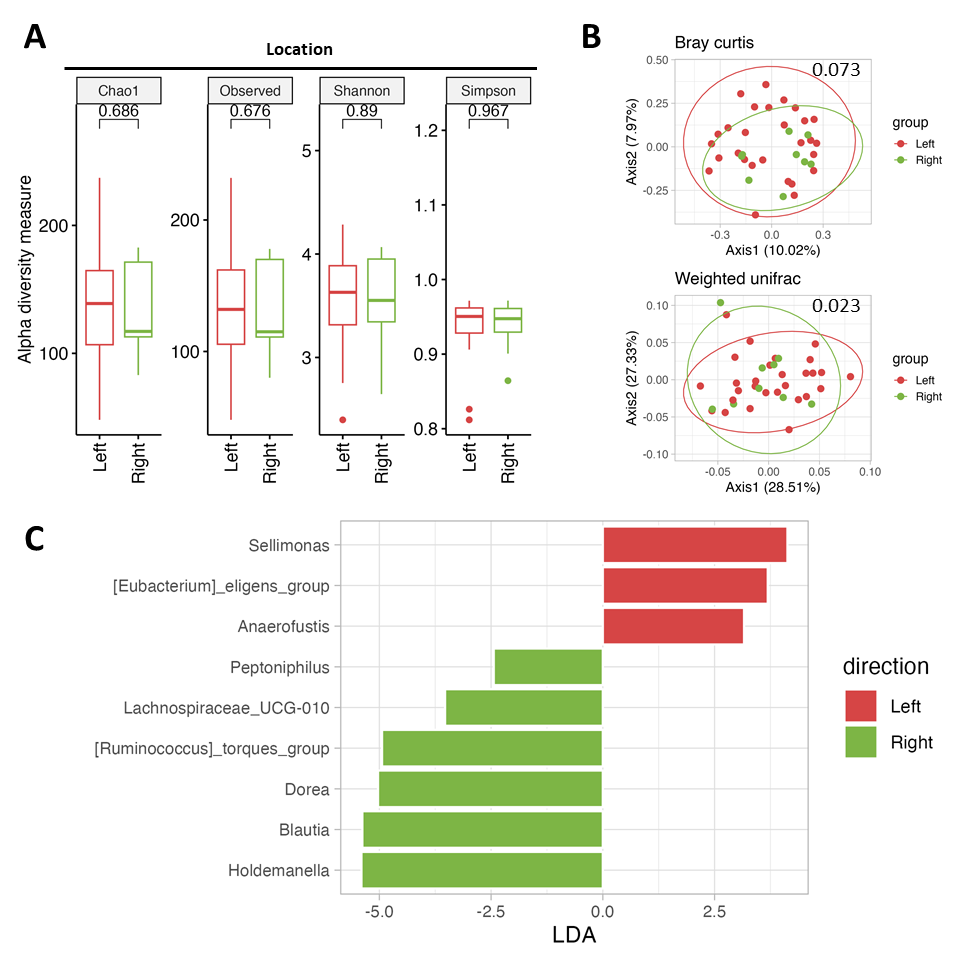


**
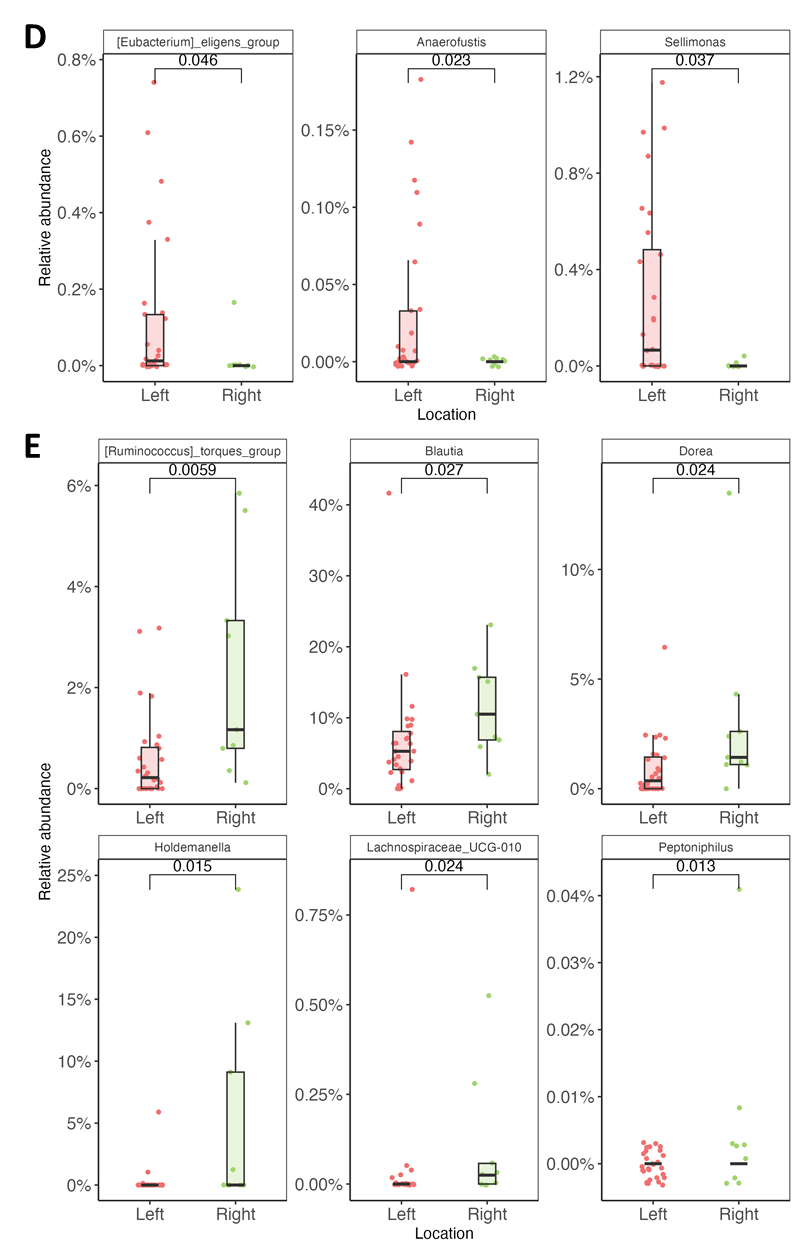
**

**Supplementary figure 4. The effect of tumor location on microbial diversity and composition**. (A) Alpha-diversity, as measured by Chao1, Observed ASVs, Shannon, and Simpson indices, is illustrated for patients in Left (red) compared to those in Right (green) sided CRC. (B) PCoA plots are shown for Bray Curtis (top) and weighted Unifrac (bottom), representing beta-diversity. Each point in these plots corresponds to a single sample, color-coded for Left (red) and Right (green) CRC groups. (C) LEfSe analysis identified significantly abundant genus-level taxa between Left (red) and Right (green) CRC groups. (D-E) Individual box plots for each sample are provided, with dots signifying differences in microorganisms as identified through the LEfSe analysis.

## Supplementary Figure 5


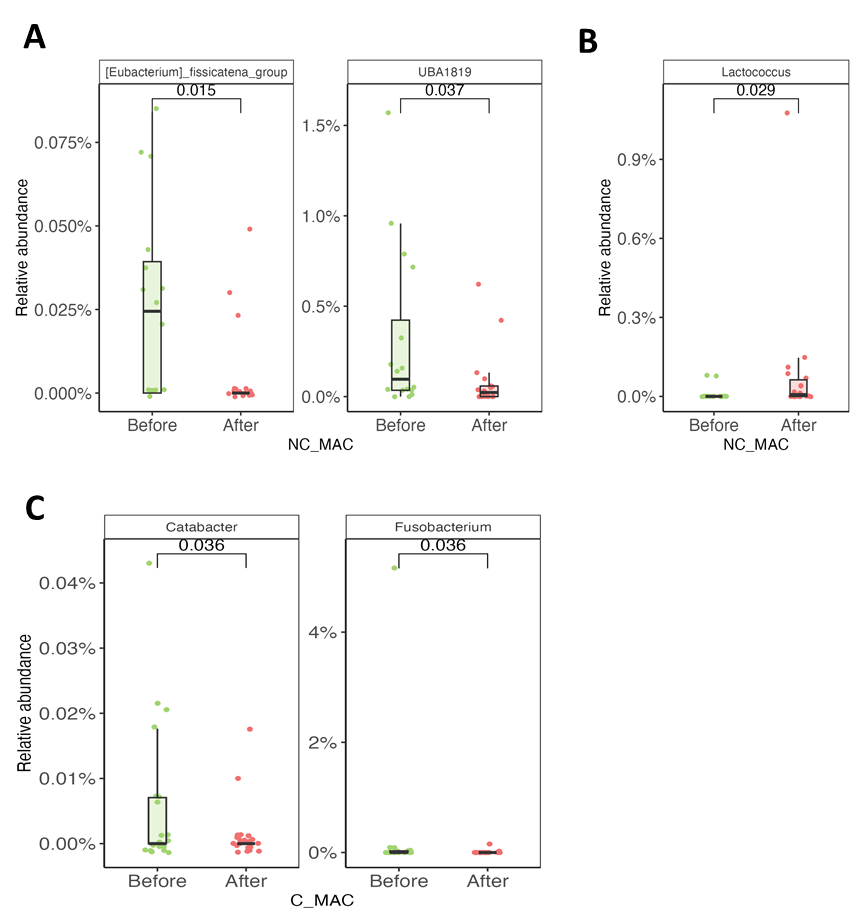


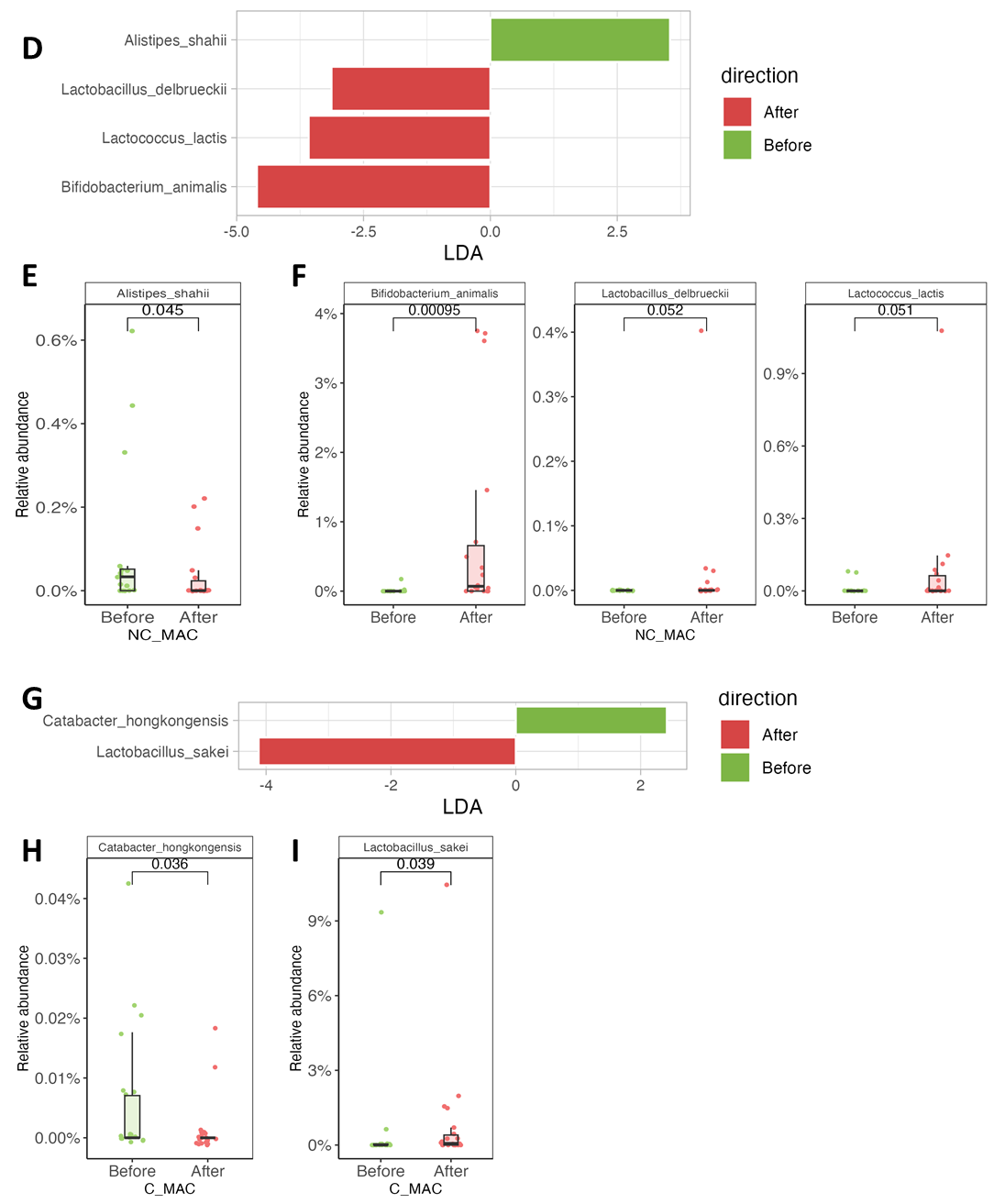


**Supplementary figure 5. The effect of smoking on microbial diversity and composition.** (A-C) Individual box plots depicted each sample with dots, illustrating the differences in microorganisms between the Before and After groups in Non-chemotherapy (A, B) and Chemotherapy cases (C), as identified through genus-level LEfSe analysis. (D) The LEfSe analysis revealed significantly abundant species-level taxa in Before (green) and After (red) mMAC diet in Non-chemotherapy group (E,F) Individual box plots represented each sample with dots, displaying differences in microorganisms as identified through species-level LEfSe analysis. (G) The LEfSe analysis revealed significantly abundant species-level taxa in Before (green) and After (red) mMAC diet in Chemotherapy group (H,I) Individual box plots represented each sample with dots, displaying differences in microorganisms as identified through species-level LEfSe analysis.

## Supplementary Figure 6


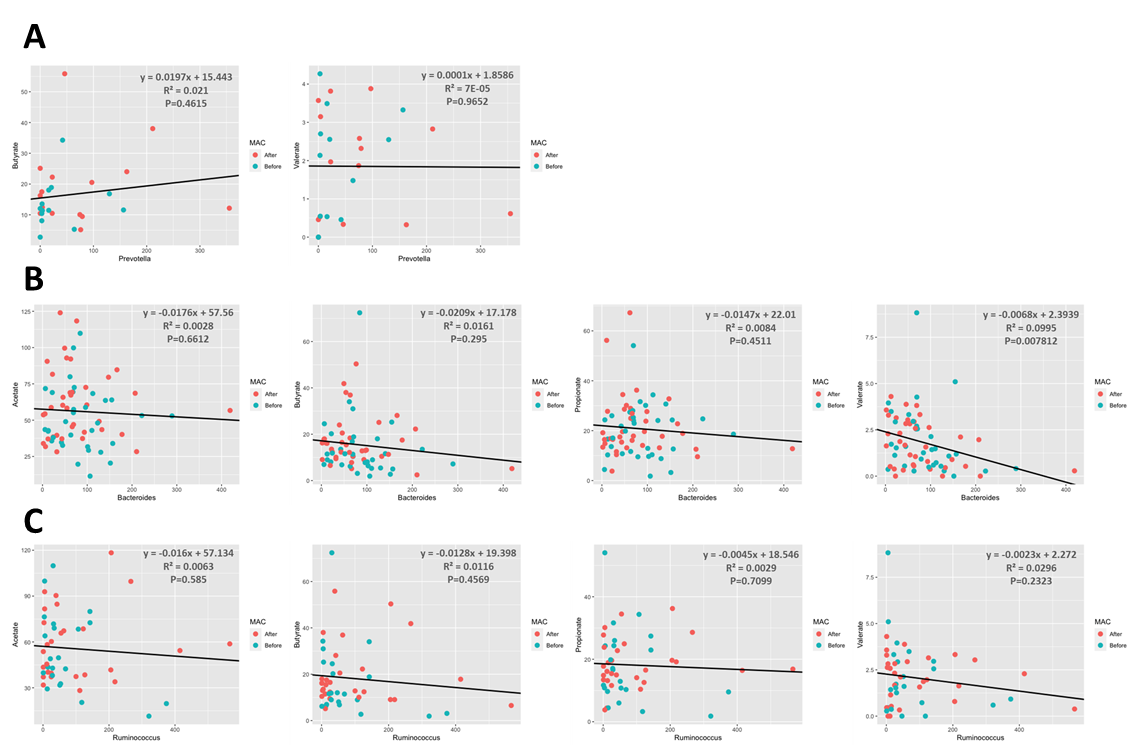


**Supplementary figure 6.** Correlation analysis of the *Prevotella, Bacteroides* and *Ruminococcus* with SCFAs (A-C) ASV count of *Prevotella* (A), *Bacteroides* (B) or *Ruminoccocus* (C) and concentration(µmol/g) of SCFAs were used for analysis. Black line represents the trend line of all samples and equation, R^2^ and p-values are indicated in each graph.

## Supplementary Figure 7


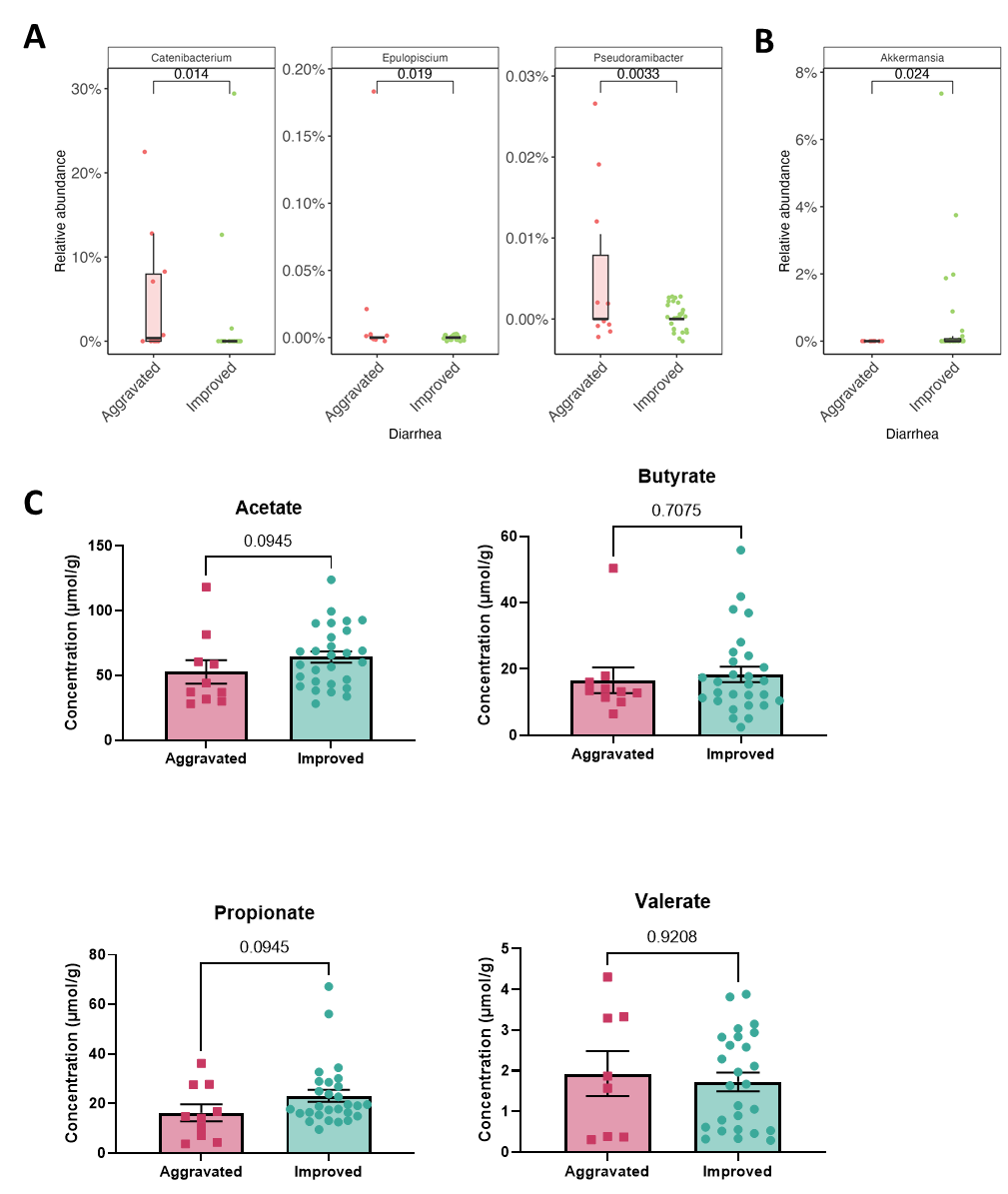


**
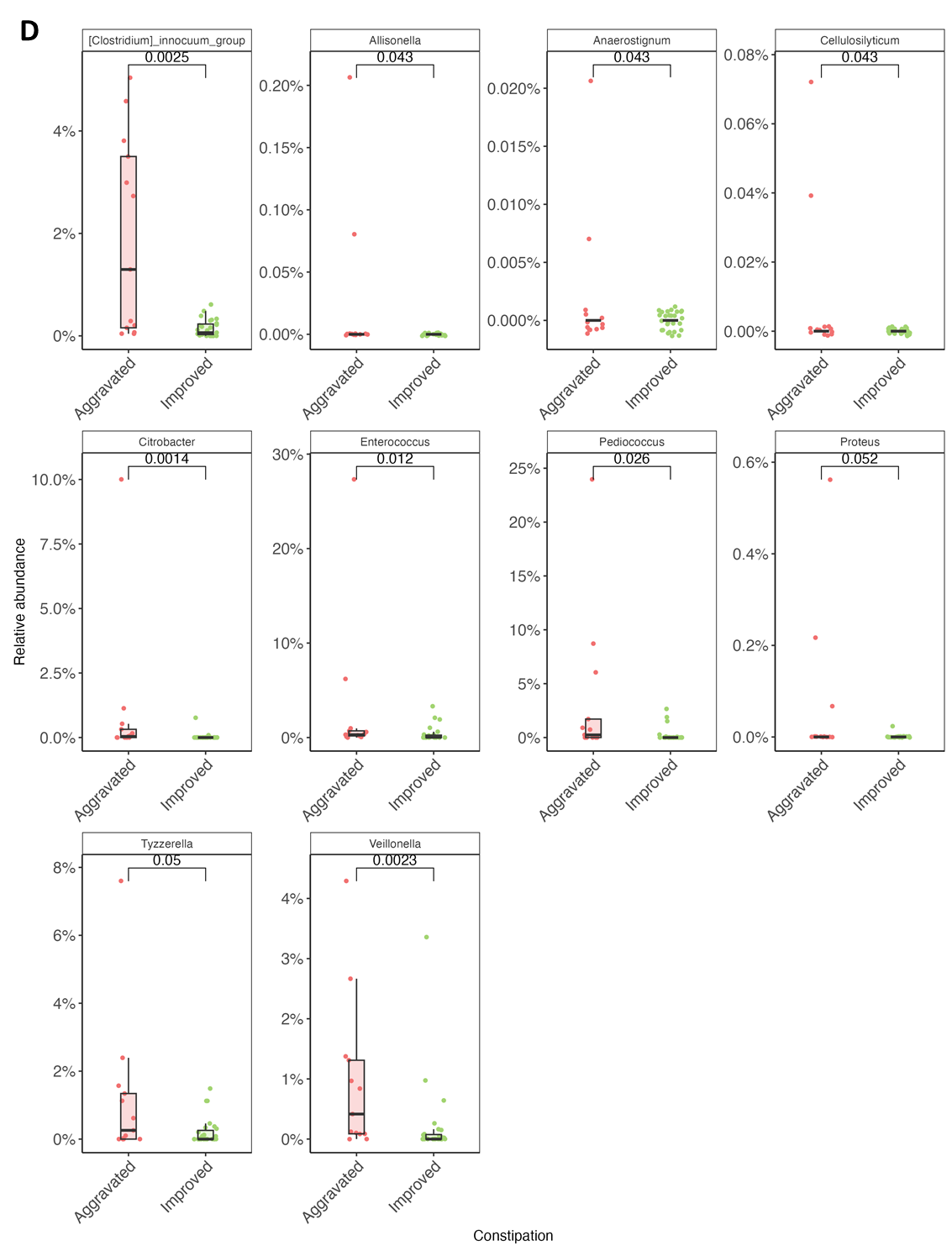
**

**
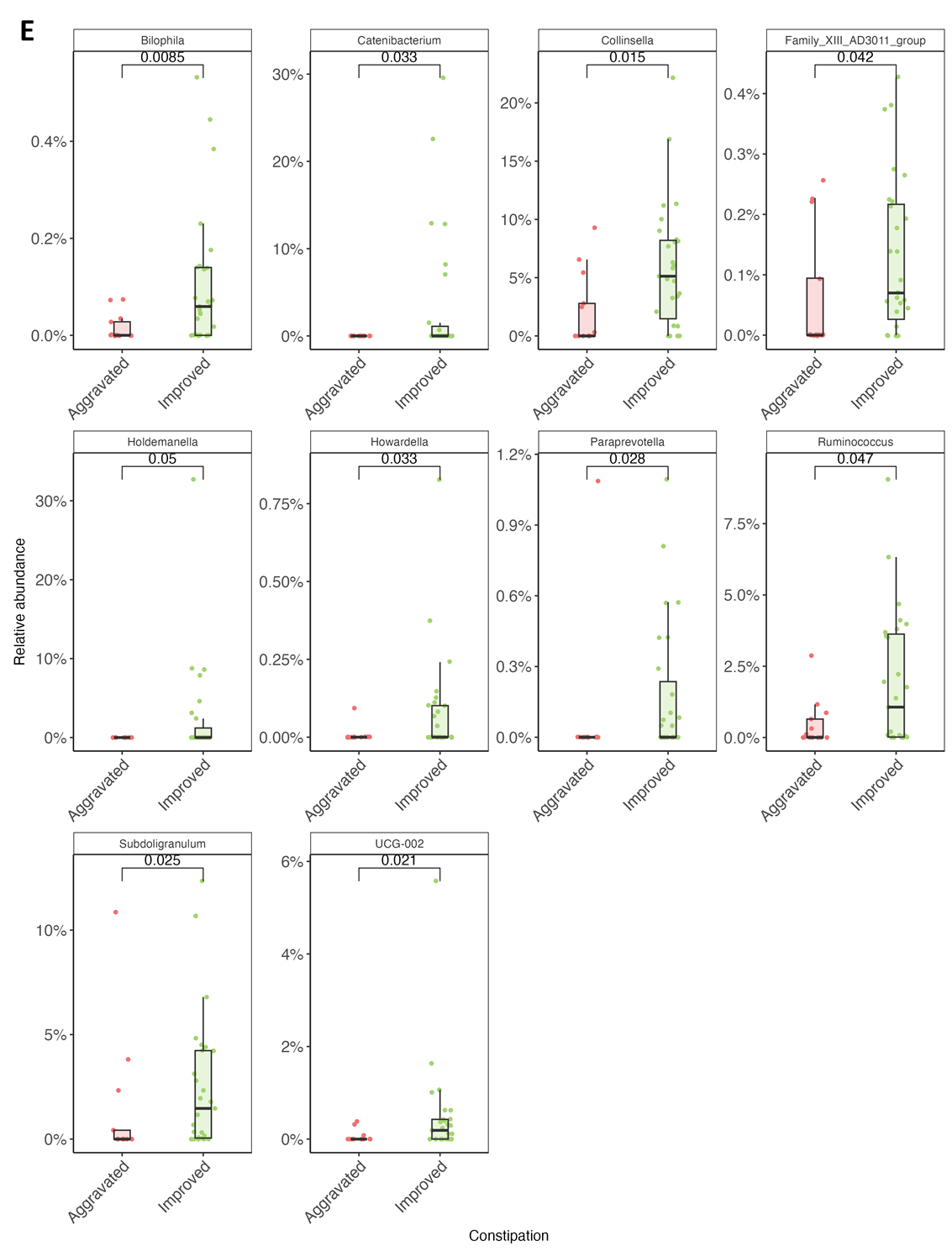
**

**
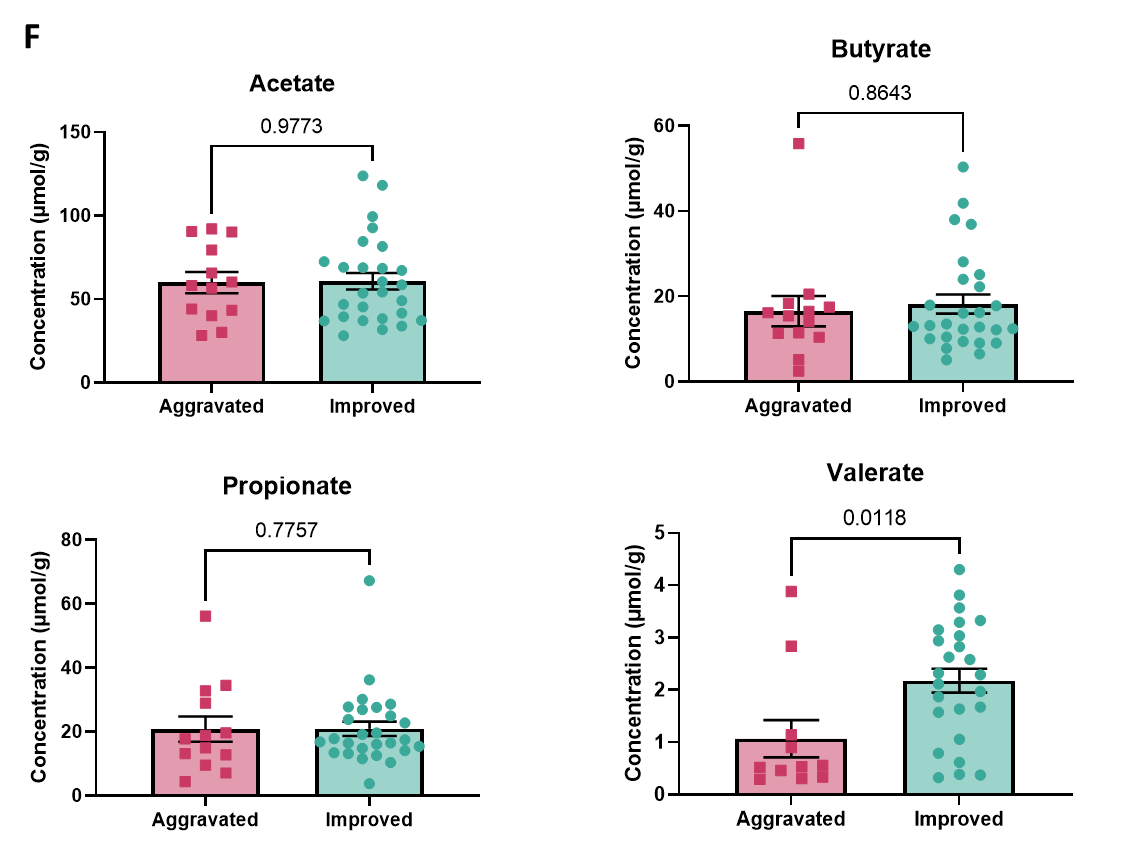
**

**Supplementary figure 7. Individual taxa plot and comparison of metabolites according to bowel habit (diarrhea and constipation).** (A,B) Individual box plots depicted each sample with dots, illustrating the differences in microorganisms between the ‘Aggravated’ and 'Improved' diarrhea groups (C) Box and whisker plots represent for acetate, propionate, butyrate and valerate levels in the aggravated and improved diarrhea groups. (D,E) Individual box plots depicted each sample with dots, illustrating the differences in microorganisms between the ‘Aggravated’ and 'Improved' constipation groups (F) Box and whisker plots represent for acetate, propionate, butyrate and valerate levels in the aggravated and improved constipation groups.

# Supplementary Table

## Supplementary table 1. Percentage of the consumed diet composition relative to the recommended daily intake.

|  | Conventional diet | | | |  | modified MAC diet | | | |
| --- | --- | --- | --- | --- | --- | --- | --- | --- | --- |
|  | average | Min | Max | SD |  | average | Min | Max | SD |
| Calorie | 85.14 | 51.65 | 120.02 | 16.95 |  | 94.30 | 50.97 | 143.43 | 19.76 |
| Protein | 90.87 | 57.91 | 140.32 | 23.08 |  | 117.92 | 59.05 | 181.15 | 30.29 |
| Fiber | 88.88 | 45.55 | 174.88 | 27.95 |  | 107.31 | 55.14 | 181.11 | 26.29 |
| Fat | 77.59 | 37.86 | 125.00 | 20.14 |  | 89.67 | 41.96 | 138.61 | 24.42 |
| Sodium | 171.34 | 75.93 | 304.37 | 49.45 |  | 158.40 | 58.80 | 266.83 | 45.94 |
| Cholesterol | 96.93 | 37.92 | 174.62 | 37.98 |  | 107.09 | 41.39 | 189.09 | 41.44 |

## Supplementary table 2. Example of modified MAC diet

| **Week 1** | | **Monday** | **Tuesday** | **Wednesday** | **Thursday** | **Friday** | **Saturday** | **Sunday** |
| --- | --- | --- | --- | --- | --- | --- | --- | --- |
| **Breakfast** | **Rice** | mixed grain rice | mixed grain rice | mixed grain rice | mixed grain rice | mixed grain rice | mixed grain rice | mixed grain rice |
|  | **Side dish** | Autonomous intake (including 100g of protein foods such as eggs, tofu, fish, and seafood excluding red meat) | | | | | | |
|  | **Kimchi** | Autonomous intake (1 bowl, 40-45g) | | | | | | |
| **Lunch** | **Lunch box** | Seafood bowl | Chicken Curry | Mapo tofu | Grilled Fish | Bibimbap | Garlic chicken ribs | Mushroom bulgogi |
| **Dinner** | **Rice** | mixed grain rice | mixed grain rice | mixed grain rice | mixed grain rice | mixed grain rice | mixed grain rice | mixed grain rice |
|  | **Meal kit** | Beef soup | Stir-fried eggplant and vegetables | Pork and vegetable stew | Braised chicken | Soft tofu soup | Soybean paste  stir-fried vegetables | Soft tofu  mixed vegetables |
|  | **Kimchi** | Autonomous intake (1 bowl, 40-45g) | | | | | | |
| **Snack** | **Fruit** | Autonomous intake (1 fist, 200~250g) | | | | | | |
|  | **Protein bar**  **(flavor)** | Blueberry | Earl gray | Green tea | Blueberry | Earl gray | Blueberry | Green tea |
|  | **Drink** | Almond milk | Fruit  flavored drink | Yogurt | Yogurt drink | Almond milk | Yogurt | Yogurt drink |
| **Week 2** | | **Monday** | **Tuesday** | **Wednesday** | **Thursday** | **Friday** | **Saturday** | **Sunday** |
| **Breakfast** | **Rice** | mixed grain rice | mixed grain rice | mixed grain rice | mixed grain rice | mixed grain rice | mixed grain rice | mixed grain rice |
|  | **Side dish** | Autonomous intake (including 100g of protein foods such as eggs, tofu, fish, and seafood excluding red meat) | | | | | | |
|  | **Kimchi** | Autonomous intake (1 bowl, 40-45g) | | | | | | |
| **Lunch** | **Lunch box** | Seafood bowl | Chicken Curry | Mapo tofu | Bibimbap | Grilled Fish | Garlic chicken ribs | Mushroom bulgogi |
| **Dinner** | **Rice** | mixed grain rice | mixed grain rice | mixed grain rice | mixed grain rice | mixed grain rice | mixed grain rice | mixed grain rice |
|  | **Meal kit** | Stewed fish | Seafood  stir-fried vegetables | Pork and vegetable stew | Braised chicken | Soft tofu soup | Soybean paste  stir-fried vegetables | Hot spicy chicken stew |
|  | **Kimchi** | Autonomous intake (1 bowl, 40-45g) | | | | | | |
| **Snack** | **Fruit** | Autonomous intake (1 fist, 200~250g) | | | | | | |
|  | **Protein bar**  **(flavor)** | Blueberry | Earl gray | Green tea | Blueberry | Earl gray | Blueberry | Green tea |
|  | **Drink** | Almond milk | Fruit  flavored drink | Yogurt | Yogurt drink | Almond milk | Yogurt | Yogurt drink |
| **Week 3** | | **Monday** | **Tuesday** | **Wednesday** | **Thursday** | **Friday** | **Saturday** | **Sunday** |
| **Breakfast** | Rice | mixed grain rice | mixed grain rice | mixed grain rice | mixed grain rice | mixed grain rice | mixed grain rice | mixed grain rice |
|  | **Side dish** | Autonomous intake (including 100g of protein foods such as eggs, tofu, fish, and seafood excluding red meat) | | | | | | |
|  | **Kimchi** | Autonomous intake (1 bowl, 40-45g) | | | | | | |
| **Lunch** | **Lunch box** | Chicken Curry | Seafood bowl | Mapo tofu | Garlic chicken ribs | Mushroom bulgogi | Grilled Fish | Bibimbap |
| **Dinner** | Rice | mixed grain rice | mixed grain rice | mixed grain rice | mixed grain rice | mixed grain rice | mixed grain rice | mixed grain rice |
|  | **Meal kit** | Stir-fied eggplant and vegetables | Beef soup | Pork and vegetable stew | Stewed fish | Soft tofu  mixed vegetables | Seafood  stir-fried vegetables | Hot spicy chicken stew |
|  | **Kimchi** | Autonomous intake (1 bowl, 40-45g) | | | | | | |
| **Snack** | **Fruit** | Autonomous intake (1 fist, 200~250g) | | | | | | |
|  | **Protein bar**  **(flavor)** | Blueberry | Earl gray | Green tea | Blueberry | Earl gray | Blueberry | Green tea |
|  | **Drink** | Almond milk | Fruit  flavored drink | Yogurt | Yogurt drink | Almond milk | Yogurt | Yogurt drink |

## Supplementary table 3. Microbiome analysis metadata

| id | Chemotherapy | Diet_order | Sample_point | MAC_Non-chemo | MAC_Chemo | Stage | Location | Diarrhea | Constipation |
| --- | --- | --- | --- | --- | --- | --- | --- | --- | --- |
| DC001-F1 | Non-chemotherapy | FREE-to-MAC | 3week | Before |  |  |  |  |  |
| DC001-F2 | Non-chemotherapy | FREE-to-MAC | 6week | After |  |  |  | Improved | Aggravated |
| DC001-I | Non-chemotherapy | FREE-to-MAC | Baseline |  |  | I+II | Left |  |  |
| DC002-F1 | Non-chemotherapy | FREE-to-MAC | 3week | Before |  |  |  |  |  |
| DC002-F2 | Non-chemotherapy | FREE-to-MAC | 6week | After |  |  |  | Improved | Improved |
| DC002-I | Non-chemotherapy | FREE-to-MAC | Baseline |  |  | I+II | Left |  |  |
| DC003-F1 | Non-chemotherapy | FREE-to-MAC | 3week | Before |  |  |  |  |  |
| DC003-F2 | Non-chemotherapy | FREE-to-MAC | 6week | After |  |  |  | Aggravated | Aggravated |
| DC003-I | Non-chemotherapy | FREE-to-MAC | Baseline |  |  | I+II | Right |  |  |
| DC004-F1 | Chemotherapy | FREE-to-MAC | 3week |  | Before |  |  |  |  |
| DC004-F2 | Chemotherapy | FREE-to-MAC | 6week |  | After |  |  | Improved | Improved |
| DC004-I | Chemotherapy | FREE-to-MAC | Baseline |  |  | III | Left |  |  |
| DC005-F1 | Chemotherapy | FREE-to-MAC | 3week |  | Before |  |  |  |  |
| DC005-F2 | Chemotherapy | FREE-to-MAC | 6week |  | After |  |  |  | Improved |
| DC005-I | Chemotherapy | FREE-to-MAC | Baseline |  |  | III | Left |  |  |
| DC006-F1 | Chemotherapy | FREE-to-MAC | 3week |  | Before |  |  |  |  |
| DC006-F2 | Chemotherapy | FREE-to-MAC | 6week |  | After |  |  | Improved | Aggravated |
| DC006-I | Chemotherapy | FREE-to-MAC | Baseline |  |  | III | Left |  |  |
| DC007-F1 | Non-chemotherapy | FREE-to-MAC | 3week | Before |  |  |  |  |  |
| DC007-F2 | Non-chemotherapy | FREE-to-MAC | 6week | After |  |  |  | Improved | Aggravated |
| DC007-I | Non-chemotherapy | FREE-to-MAC | Baseline |  |  | I+II | Left |  |  |
| DC008-F1 | Non-chemotherapy | FREE-to-MAC | 3week | Before |  |  |  |  |  |
| DC008-F2 | Non-chemotherapy | FREE-to-MAC | 6week | After |  |  |  | Improved | Improved |
| DC008-I | Non-chemotherapy | FREE-to-MAC | Baseline |  |  | I+II | Right |  |  |
| DC009-F1 | Chemotherapy | FREE-to-MAC | 3week |  | Before |  |  |  |  |
| DC009-F2 | Chemotherapy | FREE-to-MAC | 6week |  | After |  |  |  | Improved |
| DC009-I | Chemotherapy | FREE-to-MAC | Baseline |  |  | I+II | Left |  |  |
| DC010-F1 | Chemotherapy | FREE-to-MAC | 3week |  | Before |  |  |  |  |
| DC010-F2 | Chemotherapy | FREE-to-MAC | 6week |  | After |  |  | Improved | Improved |
| DC010-I | Chemotherapy | FREE-to-MAC | Baseline |  |  | III | Left |  |  |
| DC011-F1 | Non-chemotherapy | FREE-to-MAC | 3week | Before |  |  |  |  |  |
| DC011-F2 | Non-chemotherapy | FREE-to-MAC | 6week | After |  |  |  | Improved | Improved |
| DC011-I | Non-chemotherapy | FREE-to-MAC | Baseline |  |  | I+II | Left |  |  |
| DC012-F1 | Chemotherapy | FREE-to-MAC | 3week |  | Before |  |  |  |  |
| DC012-F2 | Chemotherapy | FREE-to-MAC | 6week |  | After |  |  | Improved | Improved |
| DC012-I | Chemotherapy | FREE-to-MAC | Baseline |  |  | I+II | Left |  |  |
| DC013-F1 | Non-chemotherapy | FREE-to-MAC | 3week | Before |  |  |  |  |  |
| DC013-F2 | Non-chemotherapy | FREE-to-MAC | 6week | After |  |  |  | Improved | Improved |
| DC013-I | Non-chemotherapy | FREE-to-MAC | Baseline |  |  | I+II | Left |  |  |
| DC014-F2 | Non-chemotherapy | FREE-to-MAC | 6week | After |  |  |  | Improved | Aggravated |
| DC014-I | Non-chemotherapy | FREE-to-MAC | Baseline |  |  | I+II | Left |  |  |
| DC015-F1 | Chemotherapy | FREE-to-MAC | 3week |  | Before |  |  |  |  |
| DC015-F2 | Chemotherapy | FREE-to-MAC | 6week |  | After |  |  | Improved | Improved |
| DC015-I | Chemotherapy | FREE-to-MAC | Baseline |  |  | III | Left |  |  |
| DC016-F2 | Non-chemotherapy | FREE-to-MAC | 6week | After |  |  |  | Aggravated | Improved |
| DC016-I | Non-chemotherapy | FREE-to-MAC | Baseline |  |  | I+II | Left |  |  |
| DC017-F1 | Chemotherapy | FREE-to-MAC | 3week |  | Before |  |  |  |  |
| DC017-F2 | Chemotherapy | FREE-to-MAC | 6week |  | After |  |  | Improved | Aggravated |
| DC017-I | Chemotherapy | FREE-to-MAC | Baseline |  |  | III | Right |  |  |
| DC018-F2 | Chemotherapy | FREE-to-MAC | 6week |  | After |  |  | Aggravated | Improved |
| DC018-I | Chemotherapy | FREE-to-MAC | Baseline |  |  | III | Right |  |  |
| DC019-F1 | Non-chemotherapy | FREE-to-MAC | 3week | Before |  |  |  |  |  |
| DC019-F2 | Non-chemotherapy | FREE-to-MAC | 6week | After |  |  |  | Improved | Improved |
| DC019-I | Non-chemotherapy | FREE-to-MAC | Baseline |  |  | I+II | Left |  |  |
| DC020-F1 | Chemotherapy | FREE-to-MAC | 3week |  | Before |  |  |  |  |
| DC020-F2 | Chemotherapy | FREE-to-MAC | 6week |  | After |  |  | Improved | Improved |
| DC021-F1 | Chemotherapy | MAC-to-FREE | 3week |  | After |  |  | Improved | Aggravated |
| DC021-I | Chemotherapy | MAC-to-FREE | Baseline |  | Before | III | Left |  |  |
| DC022-F1 | Chemotherapy | MAC-to-FREE | 3week |  | After |  |  | Aggravated | Aggravated |
| DC022-F2 | Chemotherapy | MAC-to-FREE | 6week |  |  |  |  |  |  |
| DC022-I | Chemotherapy | MAC-to-FREE | Baseline |  | Before | III | Left |  |  |
| DC023-F1 | Chemotherapy | MAC-to-FREE | 3week |  | After |  |  | Aggravated | Improved |
| DC023-F2 | Chemotherapy | MAC-to-FREE | 6week |  |  |  |  |  |  |
| DC024-F1 | Chemotherapy | MAC-to-FREE | 3week |  | After |  |  | Aggravated | Improved |
| DC024-F2 | Chemotherapy | MAC-to-FREE | 6week |  |  |  |  |  |  |
| DC024-I | Chemotherapy | MAC-to-FREE | Baseline |  | Before | III | Left |  |  |
| DC025-F1 | Non-chemotherapy | MAC-to-FREE | 3week | After |  |  |  | Improved | Aggravated |
| DC025-F2 | Non-chemotherapy | MAC-to-FREE | 6week |  |  |  |  |  |  |
| DC025-I | Non-chemotherapy | MAC-to-FREE | Baseline | Before |  | I+II | Left |  |  |
| DC026-F1 | Non-chemotherapy | MAC-to-FREE | 3week | After |  |  |  | Improved | Improved |
| DC026-F2 | Non-chemotherapy | MAC-to-FREE | 6week |  |  |  |  |  |  |
| DC026-I | Non-chemotherapy | MAC-to-FREE | Baseline | Before |  | I+II | Left |  |  |
| DC027-F1 | Chemotherapy | MAC-to-FREE | 3week |  | After |  |  | Improved | Improved |
| DC027-F2 | Chemotherapy | MAC-to-FREE | 6week |  |  |  |  |  |  |
| DC028-F1 | Non-chemotherapy | MAC-to-FREE | 3week | After |  |  |  | Improved | Aggravated |
| DC028-F2 | Non-chemotherapy | MAC-to-FREE | 6week |  |  |  |  |  |  |
| DC028-I | Non-chemotherapy | MAC-to-FREE | Baseline | Before |  | I+II | Left |  |  |
| DC029-F1 | Chemotherapy | MAC-to-FREE | 3week |  | After |  |  | Improved | Aggravated |
| DC029-F2 | Chemotherapy | MAC-to-FREE | 6week |  |  |  |  |  |  |
| DC029-I | Chemotherapy | MAC-to-FREE | Baseline |  | Before | I+II | Left |  |  |
| DC030-F1 | Chemotherapy | MAC-to-FREE | 3week |  | After |  |  | Improved | Aggravated |
| DC030-F2 | Chemotherapy | MAC-to-FREE | 6week |  |  |  |  |  |  |
| DC030-I | Chemotherapy | MAC-to-FREE | Baseline |  | Before | I+II | Left |  |  |
| DC031-F1 | Non-chemotherapy | MAC-to-FREE | 3week | After |  |  |  | Aggravated | Improved |
| DC031-F2 | Non-chemotherapy | MAC-to-FREE | 6week |  |  |  |  |  |  |
| DC031-I | Non-chemotherapy | MAC-to-FREE | Baseline | Before |  | I+II | Right |  |  |
| DC032-F1 | Non-chemotherapy | MAC-to-FREE | 3week | After |  |  |  | Aggravated | Improved |
| DC032-F2 | Non-chemotherapy | MAC-to-FREE | 6week |  |  |  |  |  |  |
| DC032-I | Non-chemotherapy | MAC-to-FREE | Baseline | Before |  | I+II | Left |  |  |
| DC033-F1 | Non-chemotherapy | MAC-to-FREE | 3week | After |  |  |  | Improved | Improved |
| DC033-F2 | Non-chemotherapy | MAC-to-FREE | 6week |  |  |  |  |  |  |
| DC033-I | Non-chemotherapy | MAC-to-FREE | Baseline | Before |  | I+II | Left |  |  |
| DC034-F1 | Chemotherapy | MAC-to-FREE | 3week |  | After |  |  | Improved | Improved |
| DC034-F2 | Chemotherapy | MAC-to-FREE | 6week |  |  |  |  |  |  |
| DC034-I | Chemotherapy | MAC-to-FREE | Baseline |  | Before | III | Left |  |  |
| DC035-F1 | Non-chemotherapy | MAC-to-FREE | 3week | After |  |  |  | Improved | Improved |
| DC035-F2 | Non-chemotherapy | MAC-to-FREE | 6week |  |  |  |  |  |  |
| DC035-I | Non-chemotherapy | MAC-to-FREE | Baseline | Before |  | I+II | Left |  |  |
| DC036-F1 | Chemotherapy | MAC-to-FREE | 3week |  | After |  |  | Aggravated | Improved |
| DC036-F2 | Chemotherapy | MAC-to-FREE | 6week |  |  |  |  |  |  |
| DC036-I | Chemotherapy | MAC-to-FREE | Baseline |  | Before | III | Right |  |  |
| DC037-F1 | Non-chemotherapy | MAC-to-FREE | 3week | After |  |  |  | Improved | Improved |
| DC037-F2 | Non-chemotherapy | MAC-to-FREE | 6week |  |  |  |  |  |  |
| DC037-I | Non-chemotherapy | MAC-to-FREE | Baseline | Before |  | I+II | Right |  |  |
| DC038-F1 | Chemotherapy | MAC-to-FREE | 3week |  | After |  |  | Aggravated | Improved |
| DC038-F2 | Chemotherapy | MAC-to-FREE | 6week |  |  |  |  |  |  |
| DC038-I | Chemotherapy | MAC-to-FREE | Baseline |  | Before | III | Left |  |  |
| DC039-F1 | Chemotherapy | MAC-to-FREE | 3week |  | After |  |  | Improved | Improved |
| DC039-F2 | Chemotherapy | MAC-to-FREE | 6week |  |  |  |  |  |  |
| DC039-I | Chemotherapy | MAC-to-FREE | Baseline |  | Before | I+II | Right |  |  |
| DC040-F1 | Chemotherapy | MAC-to-FREE | 3week |  | After |  |  | Improved | Aggravated |
| DC040-F2 | Chemotherapy | MAC-to-FREE | 6week |  |  |  |  |  |  |
| DC040-I | Chemotherapy | MAC-to-FREE | Baseline |  | Before | III | Right |  |  |
